# Binding modes of the KRAS(G12C) inhibitors GDC-6036 and LY3537982 revealed by all atom molecular dynamics simulations

**DOI:** 10.1038/s41598-025-07532-2

**Published:** 2025-07-10

**Authors:** Renne Leini, Jonas Kapp, Kari Kopra, Tatu Pantsar

**Affiliations:** 1 School of Pharmacy, Faculty of Health Sciences, University of Eastern Finland, Yliopistonrinne 3, 70210 Kuopio, Finland; 2 Department of Biochemistry, University of Zurich, Winterthurerstrasse 190, 8057 Zurich, Switzerland; 3 Department of Chemistry, University of Turku, Henrikinkatu 2, 20500 Turku, Finland

**Keywords:** Molecular dynamics simulations, WaterMap, Nucleotide exchange, Chemical stability, Thermal stability, Medicinal chemistry, Computational chemistry, Targeted therapies, Molecular dynamics, Biochemical assays

## Abstract

Over 100 co-crystal structures of KRAS switch-II pocket (SII-P) targeting inhibitors are currently available in the RCSB Protein Data Bank. These publicly available structures are invaluable tools that have led to a more in-depth understanding of KRAS SII-P pocket, which is crucial for targeted drug design efforts. The binding modes (co-crystal structures) of two KRAS(G12C) inhibitors that have advanced to the clinical trials, GDC-6036 (divarasib) and LY3537982 (olomorasib), are currently unavailable. Here, we reveal the putative binding modes of these two G12C inhibitors by utilizing the available structural data of analogous compounds and molecular dynamics (MD) simulations (total simulation time of 200 micros). Our biochemical assays confirm high affinity of both inhibitors for KRAS(G12C), with differential susceptibility to typical resistance associated co-mutations. A co-mutation at Tyr96 reduces the affinity of both inhibitors, whereas a mutation at His95 negatively impacts only GDC-6036. While both inhibitors display low activity towards KRAS(WT), LY3537982 maintains high activity against other RAS isoforms with G12C mutation. As the simulation predictions align with the experimental results, our findings indicate that microsecond timescale simulations can be a valuable tool for predicting binding modes.

**Supplementary Information:**

The online version contains supplementary material available at 10.1038/s41598-025-07532-2.

## Introduction

Cryptic pockets of proteins can offer invaluable allosteric drug-targeting opportunities^1^. Although new in silico approaches are emerging to identify these pockets^2,3^, more labor-intensive efforts are usually required for their detection. The latter was the case with KRAS, one of the key targets in cancer drug discovery^4^. KRAS is a small GTPase protein that regulates activation of multiple cellular signaling pathways, including the MAPK pathway^5^. Oncogenic *KRAS* mutations result in a hyperactivated protein, and mutant KRAS is a key driver in many cancers, including pancreatic ductal adenocarcinoma (PDAC), colorectal cancer (CRC) and lung adenocarcinoma (LUAD)^4^. In addition, particular KRAS mutations are associated with specific development syndromes called RASopathies^6^. Decades of significant effort eventually resulted in the 2013 breakthrough by Shokat’s group, identifying a druggable pocket that is not fully observable in KRAS crystal structures without ligand-induced changes^7^. This pocket is located beneath the switch-II region of KRAS and was hence named as the switch-II pocket (SII-P). KRAS is a highly dynamic protein, which is especially true when considering the highly flexible switch-II region^8^. Therefore, the over 100 co-crystal structures of KRAS in complex with a SII-P small molecule binder available at the RCSB Protein Data Bank (PDB)^9^ have provided invaluable information on the dynamic behavior of this pocket. To date, efforts to target SII-P have resulted in the approval of two KRAS(G12C)-specific drugs, sotorasib and adagrasib^10,11^, while several inhibitors have entered in clinical trials^12^.

Various pharmaceutical companies have significantly contributed to the available KRAS knowledgebase by depositing several co-crystal structures of the KRAS in complex with SII-P binding small molecules to the PDB (Table 1). To date a total of 77 KRAS SII-P binder co-crystal structures have been deposited to the PDB by pharmaceutical companies. The top contributors (measured by the quantity of the deposited structures) are Boehringer-Ingelheim (15), followed by the companies with clinically approved drugs: Amgen (12) and Mirati therapeutics (12), accompanied by AstraZeneca (8), Genentech (6) and Novartis (5) among others.

**Table 1:** Pharmaceutical companies that have contributed to the publicly available KRAS SII-P small molecule binder co-crystal structures (situation on May 16, 2025).

Company	No. of released structures	PDB IDs
Amgen	12	6P8W; 6P8X; 6P8Y; 6P8Z; 6OIM; 6PGO; 6PGP; 8DNI; 8DNJ; 8DNK; 9E9H; 9E9I
Array Biopharma & Mirati therapeutics	12	6N2J; 6N2K; 6USX; 6UT0; 6USZ; 7RPZ; 7RT1; 7RT2; 7RT3; 7RT4; 7RT5; 7T47
Astellas Pharma	3	7YCC; 7YCE; 8X6R
AstraZeneca	8	6T5B; 6T5U; 6T5V; 7OO7; 7O83; 7O70; 8B6I; 8B78
Bayer	2	6TAM; 6TAN
Boehringer-Ingelheim	15	8AFB; 8AFC; 8AFD; 7U8H; 8AZZ; 8AZX; 8AZR; 8AZY; 8B00; 8AZV; 8ONV; 8QUG; 8QW6; 8QW7; 8QVU; 9GBJ
BridgeBio	2	8V39; 8V3A
Genentech	6	7MDP; 7RP3; 7RP4; 8UN3; 8UN4; 8UN5
Erasca	3	8TXE; 8TXG; 8TXH
Incyte Corporation	2	9E5F; 9E5D
Jacobio Pharmaceuticals	2	9KPM; 9KPN
Merck	1	8S8C
Novartis	5	7R0M; 7R0N; 7R0Q; 8AQ5; 8AQ7
Wellspring Biosciences (Kura Oncology, Janssen)	4	5F2E; 5V9U; 6B0V; 6B0Y

**Fig. 1:**
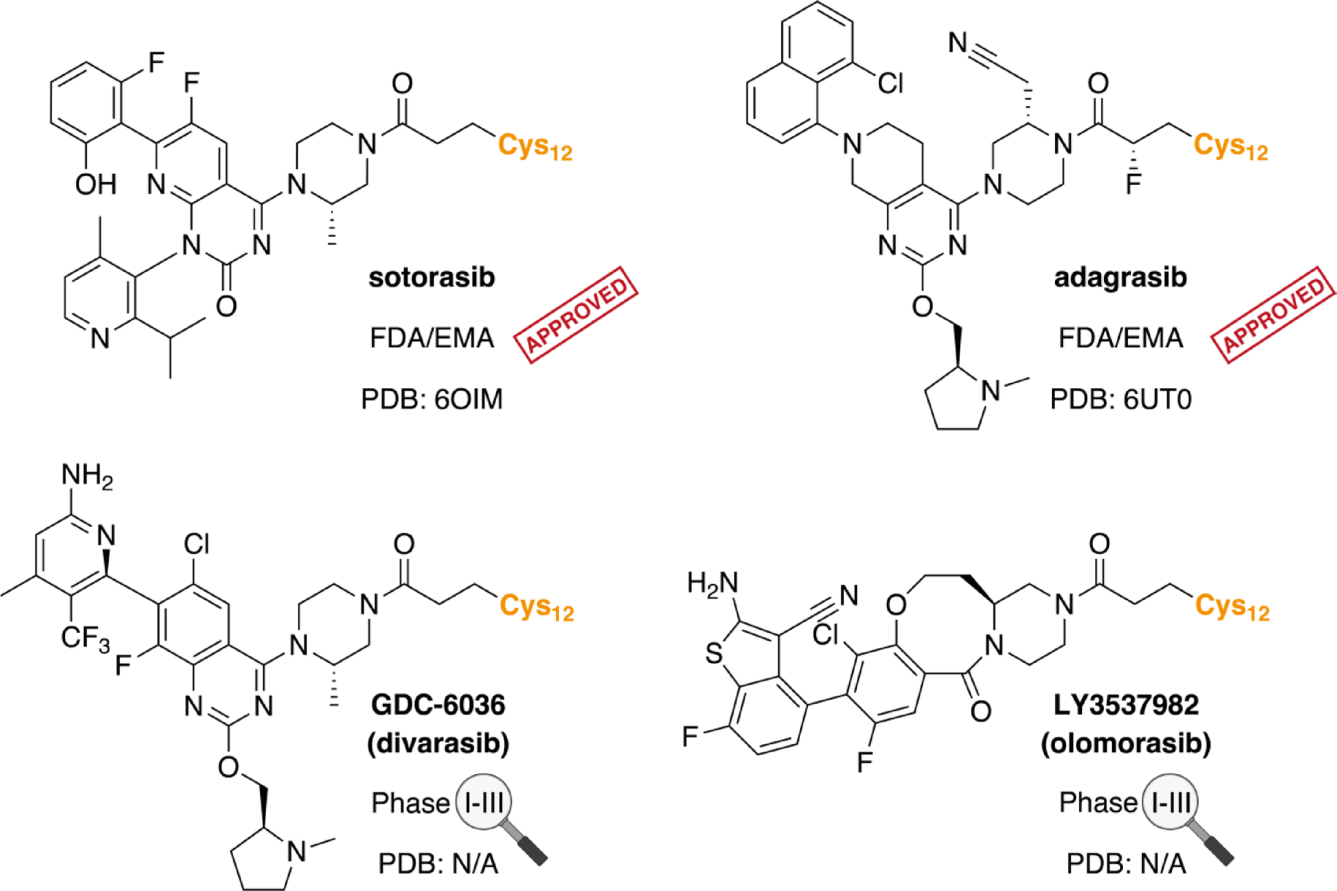
Structures of selected KRAS(G12C) bound inhibitors in their Cys12 associated configuration. Co-crystal structures of the clinically approved inhibitors KRAS(G12C)–sotorasib (PDB ID: 6OIM) and KRAS(G12C)–adagrasib (PDB ID: 6UT0) are available at the RCSB Protein Data Bank. The co-crystal structures of GDC-6036 (divarasib) and LY3537982 (olomorasib), which have advanced in Phase III clinical trials, have not been publicly disclosed.

The first company to disclose publicly a KRAS SII-P binder co-crystal structure was Wellspring Biosciences with their ARS-853 (PDB ID: 5F2E) in 2016^13^. Next, they (together with Janssen and Kura Oncology) released the co-crystal structure of the first in vivo active compound, ARS-1620 (5V9U)^14^. Finally, Wellspring Biosciences released structures of two additional compounds ARS-107 and ARS-917 (6B0V; 6B0Y)^15^. In 2018, Array BioPharma in collaboration with Mirati Therapeutics disclosed two additional co-crystal structures (6N2J; 6N2K)^16^. Amgen then released the co-crystal structure of sotorasib (Fig. 1) in November 2019 (6OIM)^17^. Additional four structures of the compound series were published earlier that year (6P8W; 6P8X; 6P8Y; 6P8Z)^18^, followed by two new structures (6PGO; 6PGP)^19^. AstraZeneca released three structures (6T5B; 6T5U; 6T5V) in 2020^20^, followed by three additional compounds two years later (7OO7; 7O83, 7O70), including AZD4625 (7O70)^21^. Bayer also contributed towards the public knowledgebase with two structures (6TAM; 6TAN) in 2020^22^. In the same year, Array Biopharma and Mirati Therapeutics released three KRAS structures (6USX; 6UT0; 6USZ), including the co-crystal structure of adagrasib (Fig. 1) (6UT0)^23^. In 2022, Genentech released three CLAMP-technique related structures (7MDP; 7RP3; 7RP4)^24^. Mirati then released seven KRAS(G12D) structures, including the co-crystal structure of MRTX1133 that is currently in clinical trials (7RPZ; 7RT1; 7RT2; 7RT3; 7RT4; 7RT5; 7T47)^25,26^. Novartis joined the race with three complexes including their clinical candidate JDQ443 (7R0M; 7R0N; 7R0Q)^27^, followed by two additional KRAS structures in the series (8AQ7; 8AQ5)^28^. Astellas Pharma also released two of their structures (7YCC; 7YCE)^29^ in 2022. Amgen subsequently disclosed three co-crystal structures of G12C inhibitors developed by Araxes Pharma, AstraZeneca, and Taiho (8DNI; 8DNJ; 8DNK)^30^. Boehringer-Ingelheim published their first structures in 2022. Initially, they released four structures (7U8H; 8AFB; 8AFC; 8AFD)^31^, and more recently, co-crystal structures of the non-covalent pan-KRAS inhibitor BI-2865 in complex with KRAS WT, G12C, G12D, G12V and G13D were disclosed (8AZV; 8AZX; 8AZY; 8AZZ; 8B00)^32^. This was accompanied by BI-2493 in complex with KRAS(G13D) (8ONV), and one related precursor compound (8AZR)^32^. AstraZeneca released two additional structures in 2023, including the CNS penetrating AZD4747 (8B6I; 8B78)^33^. Erasca published G12D inhibitors with three co-crystal structures, including their lead compound ERAS-5024 (8TXE; 8TXG; 8TXH)^34^. Furthermore, Boehringer-Ingelheim in collaboration with the Ciulli Laboratory reported SII-P utilizing pan-RAS degraders^35^; they published the co-crystal structure of the lead compound used to guide the linker design (8QUG) along with three ternary complexes of the degraders (8QW6; 8QW7; 8QVU). Genentech reported their design efforts to target KRAS(G13D) with reversible inhibitors, including three co-crystal structures of their series binding to SII-P (8UN3; 8UN4; 8UN5)^36^. In 2024, Astellas pharma released the co-crystal structure of ASP6918 in complex with KRAS(G12C) (8X6R)^37^. Finally, the most recent big pharma company to contribute to the structural zeitgeist around KRAS SII-P binders was Merck, by publishing the co-crystal structure of the KRAS(G12C) covalent inhibitor MK-1084, which is currently in Phase I and Phase III (in combination with pembrolizumab) trials (8S8C)^38^. Recently, BridgeBio released their KRAS(G12C) on/off targeting BBO-8520 (in Phase I) in complex with KRAS(G12C) bound to GTP (analog) and GDP (8V39; 8V3A)^39^. Incyte corporation reported two co-crystal structures of their KRAS(G12D) inhibitors (9E5F; 9E5D), from a series finally resulting to an orally active INCB159020^40^. Amgen released their two KRAS(G12C) targeting inhibitor co-crystal structures related to a DNA-encoded library screening approach (9E9H; 9E9I)^41^. Jacobio Pharmaceuticals published two co-crystal structures, one of their KRAS(G12C) targeting leads and JAB-21822 that is in clinical trials (9KPM; 9KPN)^42^. The final contribution to this list (by 16 May 2025) was conducted by Boehringer-Ingelheim, with their X-ray crystal structure of KRAS(G12D) targeting covalent inhibitor “compound 33” (9GBJ)^43^.

Two inhibitors targeting KRAS(G12C), GDC-6036 (divarasib)^44^ and LY3537982 (olomorasib)^45^ (Fig. 1), are currently evaluated in clinical trials. Divarasib has advanced to Phase III clinical trials for non-small cell lung cancer (NSCLC) and is evaluated in multiple Phase I/II trials, additionally including patients with metastatic colorectal cancer (CRC) and other advanced or metastatic solid tumors carrying a KRAS(G12C) mutation. To date, results from two Phase I trials of divarasib have been reported^46,47^. LY3537982 (olomorasib) is currently being studied in patients with NSCLC (Phase III), CRC and other solid tumors. Here only some interim efficacy and safety results have been reported^48–50^. The discovery of GDC-6036 was disclosed in the AACR annual meeting in 2022, where an illustration of an X-ray co-crystal structure of the complex was shown^51^. The in vitro and in vivo anti-tumor activity of LY3537982 was disclosed at the AACR annual meeting in 2021, without revealing its binding mode^48^. In addition, two studies related to in vivo target engagement of GDC-6036 by mass spectrometry have been published^52,53^, while similar literature is unavailable for LY3537982. Despite both inhibitors have advanced to Phase III clinical trials, no publicly available co-crystal structures exist for either of them in the PDB (situation on May 16, 2025)^54^.

Since the co-crystal structures of GDC-6036 and LY3537982 are not available, we decided to investigate the putative binding modes of these two compounds by utilizing the available structural information of SII-P binders and microsecond timescale molecular dynamics (MD) simulations (a total of 100 μs for each compound). Nowadays, simulations of this timescale can be completed in a relatively reasonable time, if the systems are of reasonably small size (as in the case of KRAS) and if sufficient computational resources are available (~ 11 days in our case; see methods). Furthermore, we evaluated target engagement of these two inhibitors in selected biochemical assays, including nucleotide exchange, chemical stability and thermal stability assays. In this context, we investigated the compounds specificity for its cognate target KRAS(G12C), over KRAS(WT), and the KRAS G12C mutants of the RAS isoforms HRAS and NRAS. We also evaluated the compounds activity against specific double mutants that are associated with acquired resistance of the already approved KRAS(G12C) inhibitors sotorasib and adagrasib. The results from these assays support the putative binding modes predicted by the MD simulations. Our results highlight the key dynamic interactions of these compounds in the SII-P, thereby providing new insights and understanding on the dynamic behavior of SII-P and its implications in drug development.

## Results

### Binding mode and key-interactions of GDC-6036

Since the 3D coordinates of the KRAS(G12C)–GDC-6036 complex are not publicly available, we utilized the existing KRAS(G12C) co-crystal structures of analogous compounds to guide our search for the initial starting configuration, enabling us to simulate the binding mode of GDC-6036. To this end, we utilized adagrasib (PDB: 6UT0), ARS-1620 (5V9U) and sotorasib (6OIM), all of which share structural features with GDC-6036 (Fig. S1). The protein conformation was adapted from the adagrasib co-crystal structure, and the conformation of Thr58 was reversed to its native conformation (as found in most of the co-crystal structures of SII-P binders)^9^. The obtained KRAS(G12C)–GDC-6036 complex (Fig. S2, 3D coordinates available in a supporting information), was then subjected to a total of 100 μs MD simulations, comprised of 20 independent 5 μs simulation replicas.

The root-mean-square deviation (RMSD) and root-mean-square fluctuation (RMSF) values of the KRAS(G12C) protein (Figs. S3–S5), display comparable behavior as observed in other SII-P binder all-atom MD simulation studies of the microsecond timescale^9,55,56^. Generally, GDC-6036 displays stable binding in the SII-P site in the simulations (Fig. S3). Instability of the ligand is observed in only two out of 20 simulation replicas, as indicated by the higher peaks in the ligand RMSD values (Fig. S4). The highest ligand fluctuations are observed in the flexible 1-methylpyrrolidine motif, which is located in a solvent exposed area (Fig. S3). This data provides evidence that the simulations reliably model the putative binding mode and interaction profile of GDC-6036.

**Fig. 2:**
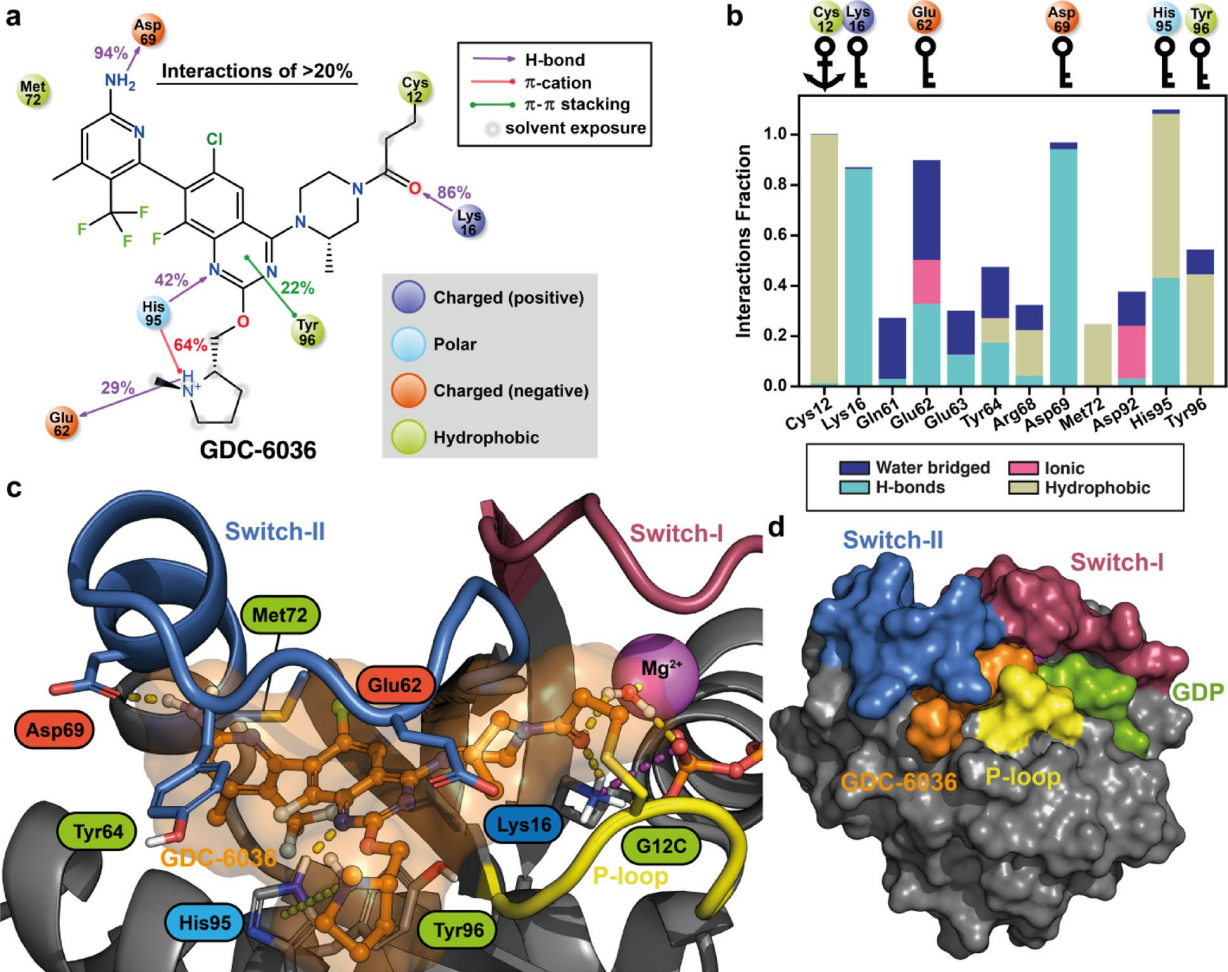
Putative binding mode of GDC-6036 to KRAS(G12C) and suggested key-interactions based on the MD simulations. (**a**) 2D-representation of the individual protein–ligand interactions observed in the MD simulations. The data consist of 100 μs simulations (20 replicas, each 5 μs long), analyzed at 1 ns intervals. Interactions observed > 20% are displayed. (**b**) A bar plot highlighting the residues that display total interactions fraction > 0.2 to GDC-6036 in the 100 μs MD simulations. Key-residues (> 0.5 interaction fraction) are highlighted with key symbols. The covalently targeted Cys12 is highlighted with an anchor symbol. (**c**) A representative snapshot from the simulations that displays the putative binding mode of GDC-6036. The snapshot was selected based on trajectory clustering and key-residue interactions (see methods). (**d**) Surface representation of the putative binding mode of GDC-6036.

The simulation data suggests that GDC-6036 forms its key interactions with Lys16, Glu62, Asp69, His95 and Tyr96 (Fig. 2; Fig S6). H-bond interactions can be observed between Lys16 and the compound’s carbonyl oxygen, a common interaction for inhibitors containing the acrylamide warhead. His95 of the α3-helix is forming H-bond interaction to the quinazoline of GDC-6036, as well as π–cation interaction to its positively charged 1-methylpyrrolidine motif. This positively charged latter moiety is also interacting with Glu62. Tyr96, located at the base of the SII-P, forms hydrophobic interactions with GDC-6036, including π–π stacking with the quinazoline. On the α2-helix side, a highly stable H-bond (94%) between Asp69 and the amino group of the 6-amino-4-methyl-3-(trifluoromethyl)pyridine-2-yl moiety is observed throughout the simulations. This amino group also exhibits occasionally H-bond interactions to the backbone Glu63 (13%), while the aromatic N of the pyridine contributes to the water mediated interactions (16%) to the same residue (Fig. 2c). Adjacent to the pocket, Tyr64 appears to shield GDC-6036 from solvent exposure by introducing its side chain as a lid on top of the inhibitor (Fig. 2c,d; Fig. S7). A representative snapshot of the putative binding mode of GDC-6036 is shown in Fig. 2c, and its 3D coordinates are available in the supporting information.

### Binding mode and key-interactions of LY3537982

Next, we moved on to predict the exact binding mode of LY3537982. Although AstraZeneca’s AZD4625 and “compound 28” (PDB: 7O70, 7OO7) share structural similarities with the core of LY3537982, we decided to use BI-0474 (8AFB) as the main guide for the starting configuration of our simulations (Fig. S8). BI-0474 carries a similar fused-thiophene headgroup when compared to LY3537982 that is bound to the α2-helix side, while the AstraZeneca compounds feature quite distinct moieties in this location. KRAS SII-P binders often exhibit atropisomerism, a feature noted in several compound series^20,28,57^. GDC-6036’s active atropisomer and its synthetic route are well-described^58,59^, but this is not the case for LY3537982. We chose the atropisomer with the chlorine atom pointing inward for our simulations, guided by analogous structures and the lack of a reasonable pose for the other isomer. This choice was later validated by the conducted biochemical assays, which confirmed the compound’s activity in this configuration. Finally, the obtained KRAS(G12C)–LY3537982 complex (Fig. S9, 3D coordinates available in supporting information), was subjected to a total of 100 μs MD simulations, comprised of 20 independent 5 μs simulation replicas.

The KRAS(G12C)–LY3537982 simulations appear consistent, with KRAS displaying regular behavior (Figs. S5, S10). LY3537982 resides tightly bound in the SII-P; only in one replica a momentary (< 200 ns) flip of the 2-amino-7-fluorobenzo[b]thiophene-3-carbonitrile moiety out of the pocket was observed, which is highlighted by a peak in the ligand RMSD (Fig. S10, Fig. S11). Overall LY3537982 displays low RMSF values, with highest fluctuations observed in the areas around the two carbonyls that are both solvent exposed (Fig. S10). Based on this data, the simulations are valid for the putative LY3537982 binding mode and interaction analysis.

**Fig. 3:**
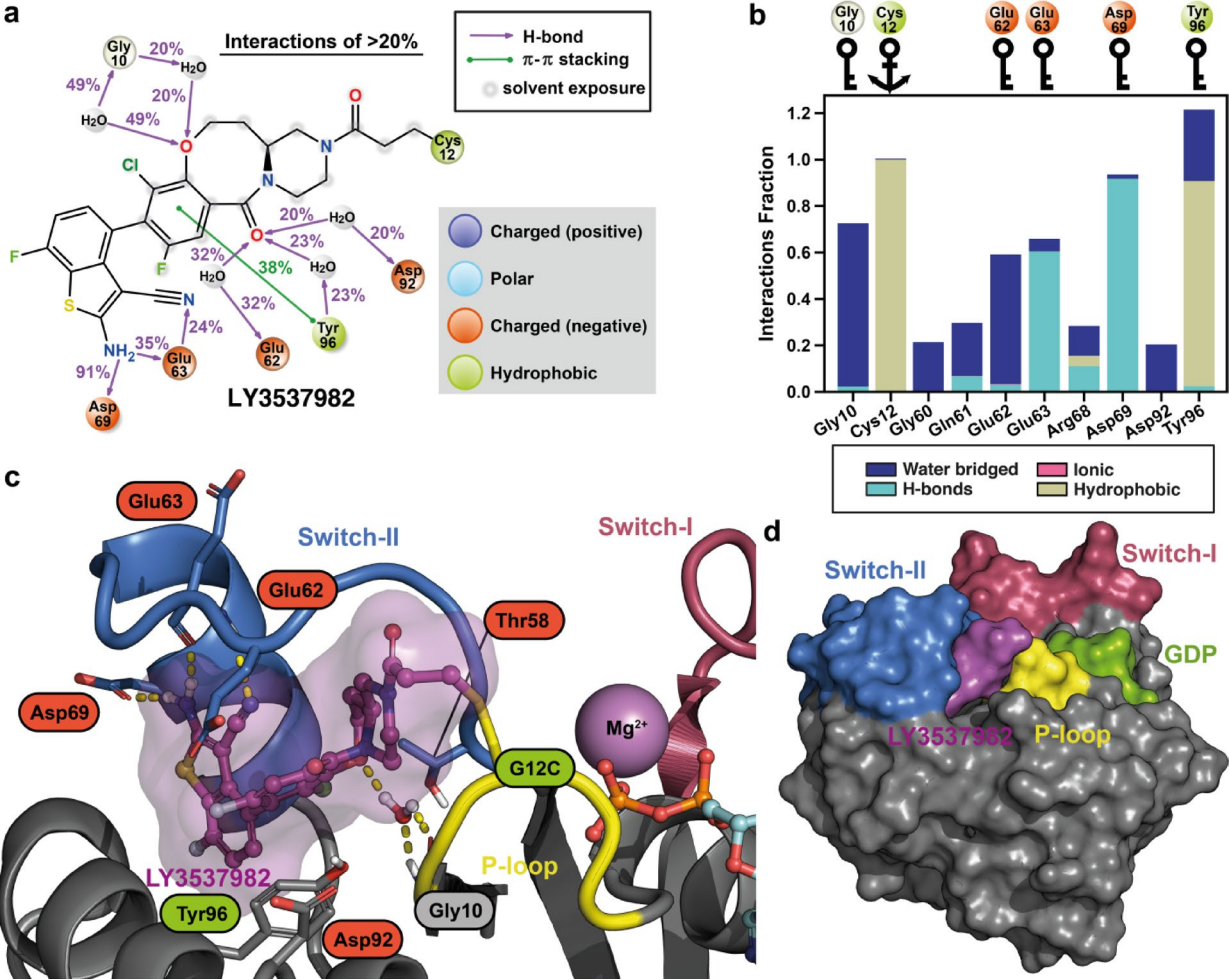
Putative binding mode of LY3537982 to KRAS(G12C) and suggested key-interactions based on the MD simulations. (**a**) 2D-representation of the individual protein–ligand interactions observed in the simulations. The data consist of 100 μs simulations (20 replicas, each 5 μs long), analyzed at 1 ns intervals. Interactions observed > 20% are displayed. (**b**) A bar plot highlighting the residues that display total interactions fraction > 0.2 to LY3537982 in the 100 μs MD simulations. Key-residues (> 0.5 interaction fraction) are highlighted with key symbols. The covalently targeted Cys12 is highlighted with an anchor symbol. (**c**) A representative snapshot from the simulations that displays the putative binding mode of LY3537982. The snapshot was selected based on trajectory clustering and key-residue interactions (see methods). (**d**) Surface representation of the putative binding mode of LY3537982.

The LY3537982 simulations suggests that the inhibitor’s key interactions occur with the residues Gly10, Glu62, Glu63, Asp69 and Tyr96 (Fig. 3; Fig. S12). Gly10 interactions are water-mediated H-bonds to the ether oxygen of LY3537982. The carbonyl oxygen of the heterocyclic ring system mediates the water-bridged interactions to polar residues in the solvent interface, including Glu62, Asp92 and Tyr96. Tyr96 further contributes to hydrophobic interactions with a relatively high frequency. The importance of the amino group of the fused-thiophene moiety is highlighted by its highly stable H-bond interaction with Asp69. The amino group is also capable of forming a H-bond with Glu63 around 35% of the time. Furthermore, Glu63 residue displays direct interactions with the nitrile group of LY3537982 (24%). Lastly, Glu62 and Tyr64 appear to close and shield the pocket from solvent (Fig. 3c,d; Fig. S7), although Tyr64 does not show direct interactions to LY3537982 frequently. In contrast to most other KRAS inhibitors, LY3537982 appears independent of H95 interactions, as reflected by the distance of H95 to the inhibitor (Fig. S7). Overall, a representative snapshot of the putative binding mode of LY3537982 is shown in Fig. 3c, and its 3D-coordinates are available in the supporting information.

### Effects of GDC-6036 and LY3537982 on Thr58-associated conserved water site

**Fig. 4:**
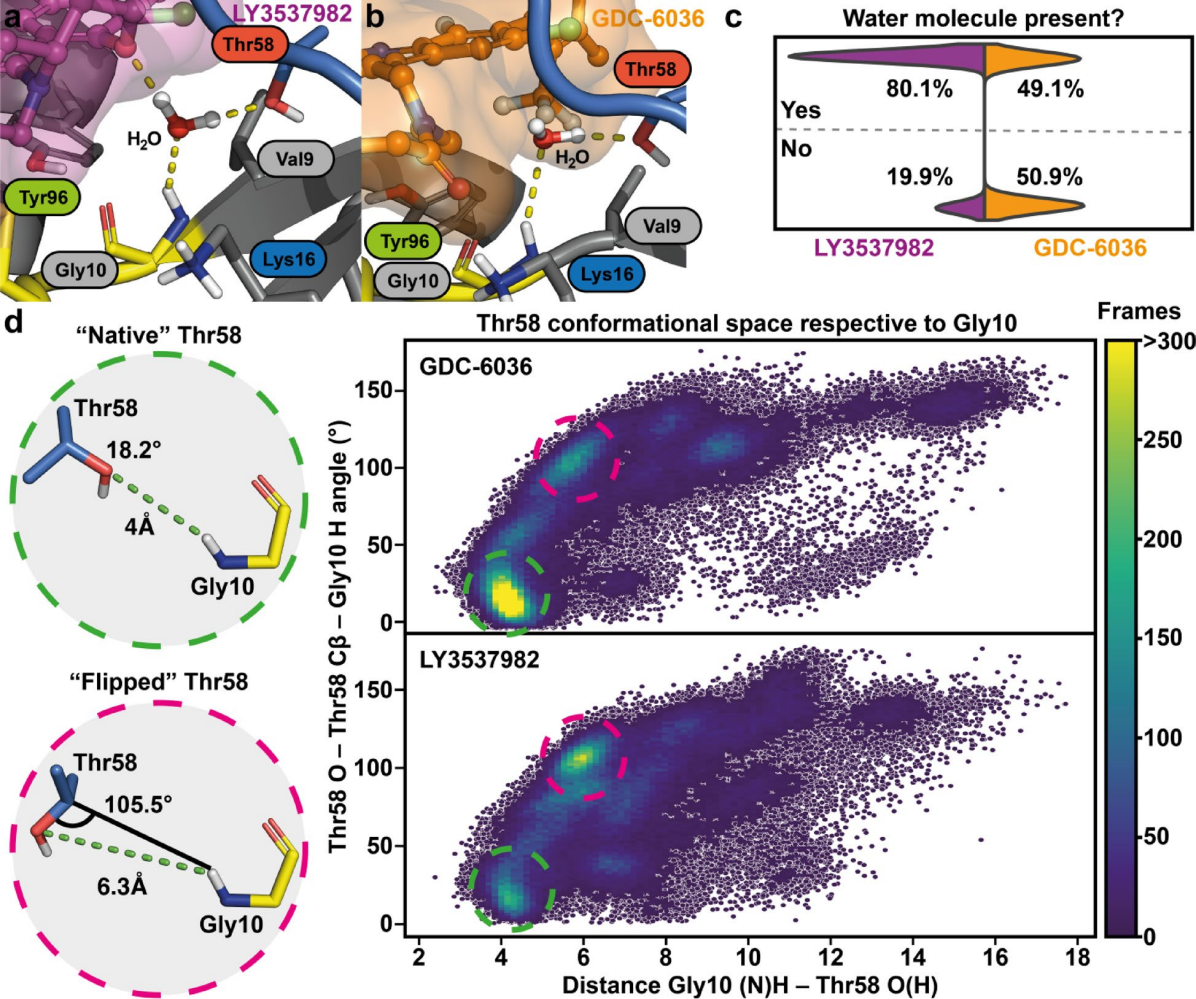
GDC-6036 and LY3537982 have different effect on Thr58-associated conserved water. (**a**) A zoom-in representation of LY3537982 and the conserved water location in a simulation snapshot. (**b**) A zoom-in representation of GDC-6036 and the conserved water location in a simulation snapshot. (**c**) Occupation of the water at the conserved water site. The water site was defined as the centroid of sidechains of residues Thr58 and Gly10. If water molecule(s) existed within 3 Å radius from the calculated centroid, water was considered to occupy the site. The data consist of 100 μs simulations (20 replicas, each 5 μs long), analyzed at 1 ns intervals. (**d**) Conformational space of Thr58 in the simulations based on the distance of its side chain oxygen to Gly10(N)H, and the angle of Thr58(O)–Thr58(Cβ)–Gly10(H). In the heatmap of the Thr58 conformational space the cells with less than 0.05 observations are transparent and individual data points are shown in dark blue.

We next investigated the impact of these two KRAS(G12C) inhibitors on the conserved water within SII-P, which is associated to Thr58 (Fig. 4a,b). This conserved water exist in numerous SII-P binder co-crystal structures and is of an interest for KRAS targeted drug discovery^9,60^. However, only a handful of SII-P binders that displace or interact with this conserved water have been reported to date^9^. The Thr58-associated water was present in this location in the starting configurations for the simulations of both compounds (Fig S13). Our simulations suggest that GDC-6036 partially promotes the displacement of the water (Fig. 4c), as it is absent in roughly half of the simulation time (50.9%). GDC-6036 lacks polar atoms near the conserved water location, which would either fully replace the water by occupying its location and interacting with Gly10 and Thr58, or introduce an attractive interaction with the conserved water (Figs. 2, 4b). In contrast, LY3537982 introduces an ether oxygen next to the conserved water location that can participate to water-mediated interactions (Figs. 3, 4a). This clearly affects the water occupation, as with LY3537982 a water molecule is observed 80.1% of the time at the expected location (Fig. 4c). Obviously, this conserved water is not static, as numerous exchange events are recorded throughout the simulations (Fig S10). Overall, water exchange is more frequent in LY3537982 simulations compared to GDC-6036 simulations, with median values of 1274 and 334 unique water molecules per replica, respectively.

Upon displacing the Thr58-associated conserved water, some compounds have been shown to impact Thr58 conformation. A “flipped” Thr58 conformation is observed in the co-crystal structures of SII-P binders that displace the water in this location with a polar nitrile group; for instance, with adagrasib (PDB: 6UT0)^9,23^ (Fig. S15). Therefore, we next analyzed the impact of these inhibitors on Thr58 itself. In the simulations of GDC-6036, Thr58 prefers the “native” conformation, which is commonly observed in KRAS crystal structures (Fig. 4d; Table S1)^9^. Interestingly, our simulations suggest that with LY3537982, for which water-bridged interactions to Gly10 are frequently observed (Fig. 3), the configuration of Thr58 is more flexible, promoting a “flipped” orientation of Thr58 (Fig. 4c). Conformation of Thr58 does not seem to explain the water occupancy difference between the two ligands. While the flipped orientation of Thr58 with GDC-6036 is associated with low water occupancy (0.03), a significantly higher occupancy (0.73) is observed in the same configuration with LY3537982 (Table S1).

We also evaluated the water energy and occupation associated with the representative conformations of the simulations (Figs. 2c, 3c) using WaterMap^61,62^. The representative conformations of both compounds show that the water exists on the site, and WaterMap suggest a high energy for the hydration site (Fig. S16; Table S2). The estimated energies for the conserved water are ΔG =  + 9.52 and + 11.37 kcal/mol for LY3537982 and GDC-6036, respectively (Fig. S16; Table S2). This is in line with the previous findings that with a SII-P binder this water site (if present) is associated with high energy^9^. However, it is unclear what is the role of this water (and its energy) for binding affinity or stability especially when the ligands are covalent inhibitors. The lower ΔG with LY3537982 is associated with an improved ΔH of the hydration site. In the representative complex configuration of LY3537982, Thr58 exist in the “flipped” orientation, i.e., the oxygen of the threonine is not accessible to the conserved water. Furthermore, additional high-energy hydration sites are observed next to Lys16 and P-loop with LY3537982 (Fig. S16), while the location of these hydration sites is occupied with the GDC-6036.

### Biochemical assays validate the high potency of GDC-6036 and LY3537982 towards KRAS(G12C)

**Fig. 5:**
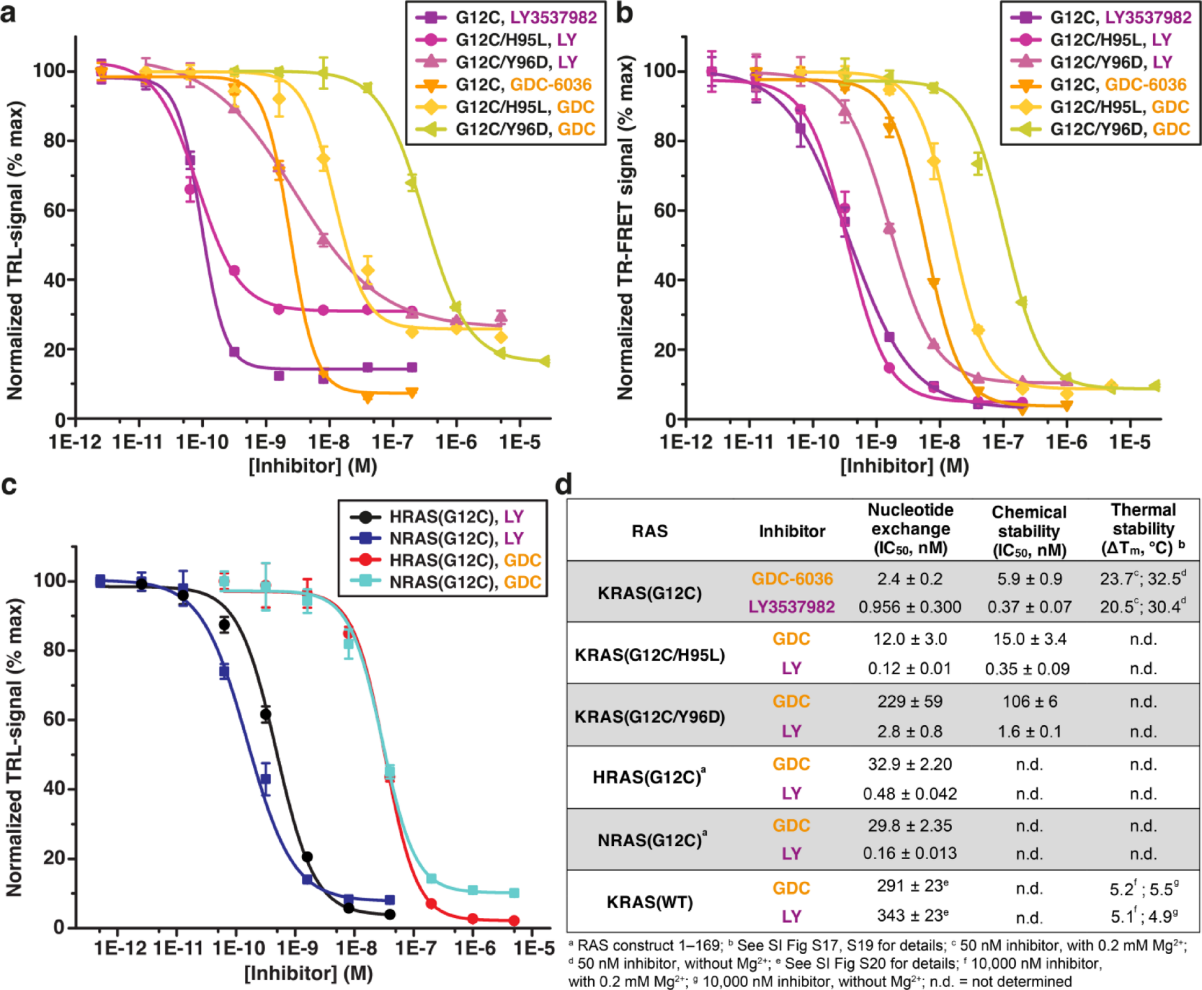
Biochemical functionality of GDC-6036 and LY3537982 in selected RAS assays. (**a**) Nucleotide exchange inhibition of KRAS(G12C), KRAS(G12C/H95L) and KRAS(G12C/Y96D) with GDC-6036 and LY3537982. Nucleotide exchange inhibition was monitored in an assay with 10 nM KRAS and 5 nM SOS^cat^, utilizing Eu^3+^-GTP TRL-signal protection upon KRAS binding according to QRET principle. (**b**) KRAS (20 nM) chemical stability in the presence of 1 M urea and varying concentration of GDC-6036 and LY3537982 was monitored using a FRET-Probe stability assay by monitoring the TR-FRET signal after 120 min incubation at room temperature. (**c**) Nucleotide exchange inhibition of NRAS(G12C) and HRAS(G12C) with GDC-6036 and LY3537982 (conditions as in **a**). (**d**) Overview of the assay results presented in (**a**–**c**) and Figs. S17, S19–S20.

Finally, we evaluated the potency of GDC-6036 and LY3537982 in selected biochemical assays (an overview of the assay results is shown in Fig. 5d). First, we performed a traditional nucleotide exchange assay using a single-label quenching resonance energy transfer (QRET) principle^63–65^ and non-labeled full-length KRAS(G12C), (G12C/H95L), and (G12C/Y96D). The selection of the two double mutants was based on the observed interactions in the MD simulations (Figs. 2, 3), and the fact that acquired resistance with SII-P inhibitors is associated with these mutations, as clinically observed for sotorasib and adagrasib^66–68^. GDC-6036 displayed a low nanomolar IC_50_ value with KRAS(G12C) in the SOS^cat^-induced nucleotide exchange (Fig. 5a), while both double mutations significantly reduced its activity. The observed IC_50_ values were: 2.4 ± 0.2 (KRAS(G12C)); 12.0 ± 3.0 (G12C/H95L); and 229 ± 59 nM (G12C/Y96D). In case of LY3537982, exceptionally low IC_50_ values of 0.956 ± 0.300 and 0.121 ± 0.005 nM were observed with KRAS(G12C) and (G12C/H95L), respectively. The values against KRAS(G12C/Y96D) were also in the low nanomolar range (IC_50_ = 2.8 ± 0.8 nM) (Fig. 5a). Due to protein concentration dependence, the IC_50_ in this assay cannot be lower than half the KRAS concentration, which is 5 nM, assuming 100% active KRAS. GDC-6036’s IC_50_ is near this theoretical minimum, while LY3537982’s IC_50_ values are substantially lower.

To further investigate the behavior of GDC-6036 and LY3537982, we changed the assay scheme to monitor KRAS stability in complex with these inhibitors. In these assays, no external protein (SOS^cat^) is used and only KRAS and inhibitors are present. First, we conducted a chemical stability assay by utilizing the FRET-Probe technique^69^. In this system, time dependent KRAS (20 nM) denaturation is monitored in 1 M urea solution after KRAS preincubation with varying inhibitor concentration. In case of GDC-6036, observed IC_50_ values are highly comparable to those observed with nucleotide exchange assay (Fig. 5b). GDC-6036 stabilized KRAS mutants in order G12C, G12C/H95L, and G12C/Y96D, and the observed IC_50_ values were 5.9 ± 0.9, 15.0 ± 3.4, and 106 ± 6 nM, respectively. To our surprise, LY3537982 displayed similar IC_50_ values as in the nucleotide exchange assay, even though the IC_50_ values in that assay were lower than the theoretical minimum. Again, IC_50_ values KRAS(G12C) and (G12C/H95L) were similarly observed in the picomolar range, 0.368 ± 0.065 and 0.345 ± 0.091 nM, and for G12C/Y96D at low nanomolar level (1.6 ± 0.1 nM), respectively (Fig. 5b). Next, we used the FRET-Probe approach^65,69^ to assess the thermal stability of KRAS(G12C) with both GDC-6036 and LY3537982 in the presence and absence of Mg^2+^ (Fig. 5d; Fig. S17). In this assay, we observed an exceptionally high stability increase with both inhibitors (Fig. 5d; Fig. S17). Thermal shift (Δ*T*_m_) of over 20 °C and 30 °C (depending on the presence of Mg^2+^) was observed with both inhibitors (at 50 nM inhibitor concentration). The data demonstrate that SOS^cat^ did not artificially lower LY3537982’s IC_50_. Furthermore, both these results and nucleotide exchange assays indicate that LY3537982’s interaction with KRAS(G12C/H95L) is comparable to its interaction with KRAS(G12C), while activity is reduced with KRAS(G12C/Y96D). In contrast, the activity of GDC-6036 is negatively impacted by both H95L and Y96D co-mutations.

SII-P inhibitor activities can differ among RAS isoforms (KRAS/HRAS/NRAS). These isoforms differ only in position 95 in the SII-P (Fig. S18). For instance, sotorasib displays activity towards NRAS(G12C) and HRAS(G12C), while adagrasib activity is compromised with these RAS isoforms due to its dependency on H95 that is only present in KRAS^68,70^. Therefore, we tested the activity of GDC-6036 and LY3537982 to HRAS(G12C) and NRAS(G12C). Aligning with the results observed with the KRAS double mutant (G12C/H95L), GDC-6036 activity is reduced for the RAS isoforms HRAS(G12C) and NRAS(G12C), while LY3537982 displays comparable IC_50_ values for all RAS isoforms (Fig. 5c,d). Notably, the predicted binding mode of LY3537982 differs clearly from the isoform agnostic sotorasib, which utilizes an isopropyl-methylpyridine to occupy a groove next to position 95 (Fig. S18).

Finally, we evaluated the potential binding of GDC-6036 and LY3537982 to KRAS(WT) present in all untransformed tissue. In a thermal stability assay, both inhibitors display comparable (low) activity towards KRAS(WT) (Fig. S19; Fig. 5d). Δ*T*_m_ values of approximately 5 °C are observed at an inhibitor concentration of 10,000 nM, with or without Mg^2+^. Furthermore, both inhibitors display submicromolar IC_50_ values with KRAS(WT) in the nucleotide exchange inhibition assay (Fig. S20; Fig. 5d), with GDC-6036 displaying marginally higher activity (GDC-6036: 291 ± 23 nM; LY3537082: 343 ± 23 nM). Our results imply that both inhibitors are highly dependent on their covalent interaction to G12C for efficient and potent RAS inhibition.

## Discussion

Our MD simulation approach, utilizing existing KRAS structural data, offers the first publicly available 3D information on the putative binding modes of the KRAS(G12C) inhibitors GDC-6036 and LY3537982. Overall, the simulations demonstrate the stable binding of both inhibitors in SII-P of KRAS(G12C), while suggesting their key-interactions in the SII-P, which are consistent with experimental results. Our biochemical assays confirm the high affinity of these compounds for KRAS(G12C) and their effect on selected KRAS double mutants. Notably, the (G12C/H95L) mutant has a negligible effect on the activity of LY3537982. Our results suggest that LY3537982 can be characterized as pan-RAS(G12C) inhibitor, while both inhibitors show a strong differentiation between mutant and wild-type KRAS.

The typical SII-P residues, such as Glu62, Asp69 and Tyr96, were identified to form key interactions with these two studied compounds, closely following observations from the analogous structures^9^. The major discrepancy in direct interactions of these two inhibitors were observed for Lys16, Glu63 and His95. The discrepancy in interactions with Glu63 and His95 can be readily explained with the structural characteristics of these compounds (Figs. 2, 3). Based on our predicted binding modes, not only is LY3537982 different to GDC-6036 regarding to its (lack of) His95 interactions, but it also appears unique when compared to the approved inhibitors adagrasib and sotorasib^68^. LY3537982 completely maintained its potency in the nucleotide exchange assay with NRAS(G12C) and HRAS(G12C). This is well-in-line with the absence of His95 interactions in its binding, which is the only residue that differs in the SII-P among RAS isoforms^68,70^. In a recent study it was also shown that the in vitro potency of GDC-6036 is compromised by the both double mutants that were also evaluated in our study (G12C/H95L) and (G12C/Y96D)^71^. However, similar data is not available for LY3537982.

Interactions between LY3537982 and Lys16 were observed with relatively low frequency, when compared to what has been observed in comparable simulations earlier with other KRAS SII-P inhibitors^9,55,68^. AstraZeneca’s “compound 28” and AZD4625 (PDB IDs: 7OO7, 7O70)^21^ that share partially analogous 8-membered ether ring structure with LY3537982 (Fig. S6), do display interaction to Lys16 in their crystal structures. The lack of this interaction could potentially be a simulation artefact that is caused by the starting configuration based on BI-0474 (PDB: 8AFB)^31^ (Figs. S8–S9), which does not display interactions with Lys16. However, identical fused-thiophene scaffold to what is present in BI-0474 (similar to that of LY3537982) is also present in the non-covalent pan-KRAS inhibitors disclosed by Boehringer-Ingelheim^32^, and these inhibitors do not rely on Lys16 interactions. In addition, the KRAS(G12C) targeting inhibitor BBO-8520 that has identical fused-thiophene moiety with LY3537982, does not display Lys16 interaction when co-crystallized with KRAS(G12C)-GNP (GTP-analogue) (PDB: 8V39)^39^. Therefore, we speculate that the fused-thiophene moiety of LY3537982 at the opposite end of the SII-P could potentially reduce the dependency on Lys16 interactions. However, it remains unclear whether the observed absence of Lys16 interactions is due to the initial simulation starting configuration, the structure of LY3537982, the simulation outcome, or a combination of these factors.

Our predicted binding modes suggest a different behavior of the Thr58-associated conserved water with these two inhibitors. While GDC-6036 appears repulsive to the conserved water and is partially capable in displacing it, LY3537982 approaches the binding differently by introducing water mediated H-bond interactions through this water and supports its existence on the location. By introducing these water-bridged interactions to Gly10, LY3537982 appears to distort the native conformation of Thr58. Overall, our biochemical data indicate that LY3537982 is more potent than GDC-6036; however, with some limitations due to maximal assay sensitivity, i.e., requirement for low nanomolar KRAS concentrations. Overall, the role of these water interactions on the overall potency remains unclear, as this is impossible to estimate with structurally different covalent inhibitors. We speculate, however, that the observed partial displacement and/or interactions with water may contribute to the compounds’ high potency.

The importance of having sufficient replicas when conducting MD simulations has been documented in the literature^72^ and emphasized in recent editorials^73,74^. This requirement has also been exemplified by our simulations, where we observed instability of the ligands in the binding pocket in 2/20 and 1/20 simulations of GDC-6036 and LY3537982, respectively. Notably, the ligands in our simulations were already optimized, high-affinity covalent inhibitors. This emphasizes that the requirements for sufficient replicas cannot be overlooked.

These putative in silico predicted binding modes of the two compounds in clinical trials provide new insights to the RAS drug discovery community. While a co-crystal structure would be preferable, these predicted binding modes offer actionable data for potential future improvements and understanding for rational drug design in the SII-P. Obtaining a high-quality crystal structure of a protein–ligand complex is not always straightforward and can be slow^75,76^, while the MD approach offers a relatively fast alternative (if experimental structures of analogous ligands and sufficient computational resources are available). During the revision of this manuscript, an academic group published the co-crystal structure of GDC-6036 (divarasib) in complex with KRAS(G12C)^77^. The MD-predicted binding mode (Fig. 2) shows strong agreement with the experimental structure, with RMSD values of 0.798 Å for the Cα atoms and 0.551 Å for the heavy atoms of GDC-6036 (Fig. S21). If and when the co-crystal structures for LY3537982 (olomorasib) is solved and released, it could further verify the accuracy of predictions achieved with unbiased MD simulations using starting configurations based on analogous structures. This is also important for accurately determining whether microsecond timescale simulations are sufficient for this type of study. This would apply to both, KRAS and structure-based drug design more broadly, determining if even longer timescales are necessary to achieve high-confidence predictions with unbiased simulations.

## Materials and methods

### Molecular modeling and molecular dynamics simulations

Molecular modeling was conducted with Maestro (Schrödinger Release 2023–1, Schrödinger LLC, New York, NY, 2023) and OPLS4 force field^78^. The co-crystal structures of the analogous inhibitors were downloaded from the PDB and prepared using Protein Preparation Wizard^79^: bond orders were assigned and hydrogens replaced, zero-order bonds to the metals were created, the missing side chains and loops were added with Prime^80,81^ and Epik^82,83^ was applied to generate the small molecule ionization states, all crystal waters were preserved, H-bonds were optimized with sampled water orientations and PROPKA, and energy minimization was conducted (0.3 Å heavy atom RMSD convergence). The aligned analogous structures were visually analyzed, and we decided to use 6UT0 and 8AFB for building our initial protein–ligand configuration. The existing ligand was replaced with studied inhibitor (adagrasib in 6UT0 with GDC-6036; BI-0474 in 8AFB with LY3537982), which was described using the default OPLS4 force field parameters, while Maestro 3D Builder was used to generate the covalent bond to the new ligand and Cys12. The initial conformation of LY3537982 was taken from the output of the QM Conformer & Tautomer Predictor (see more detailed description of the protocol in^84^). In the 8AFB structure the carbonyl group of the LY3537982 appeared to overlap with one of the crystal waters at the S-IIP (HOH: 344), which was therefore deleted. In the selected protein structures, we reverse mutated the S51; L80 and S118 back to the native cysteine residues (6UT0), and S118 in case of 8AFB. Thr2 at the N-terminus was acetylated, as well as the C-terminal residue Lys169 (6UT0) or Lys165 (8AFB). Both new complexes were run through Protein Preparation Wizard^79^, and the energy minimization was conducted twice with the 0.3 Å heavy atom RMSD convergence setting.

We used Desmond^85^ molecular dynamics engine for the simulations of the prepared systems. The energy minimized protein–ligand complexes were solvated in a cubic box with a minimum distance of 15 Å from the protein or the ligand to the edges, with K^+^- and Cl^-^ ions, to neutralize the system, and with total concentration of 150 mM. Water model TIP3P^86^ was applied. The starting configurations of the full systems are provided as SI files. Desmond simulations were run in NpT ensemble, with 1.01325 bar and 300 K (Martyna-Tobias-Klein barostat and Nosé-Hoover chain thermostat) with the default settings of RESPA integrator timesteps with 2, 2, and 6 fs applied for bonded, near and far, respectively. The cutoff for Coulombic was 9 Å. The default Desmond system relaxation protocol was applied before the production simulations. Each system was simulated for 5,000 ns with 20 replicas using a random seed, resulting in an aggregate of 100 μs simulation data for both systems. Simulations were conducted at the national supercomputer Puhti (CSC–IT Center for Science, Finland) using NVIDIA Volta V100 GPUs, which provided the simulation speed 476 ns and 452 ns/day (mean values) for LY and GDC systems, respectively. Running the simulation replicas in parallel, resulted in completion of the simulations in about 11 days.

### Simulations analysis

Protein–ligand interactions, protein RMSF and ligand RMSD were analyzed by Simulation Interaction Analysis tool (scripts: event_analysis.py; analyze_simulation.py) (Schrödinger LLC). The default settings were used in the definition of the interactions, where the following parameters were applied: H-bonds: distance of 2.5 Å between the donor and acceptor with ≥ 120° and ≥ 90° for donor and acceptor angles, respectively; π–cation interactions: 4.5 Å distance between the positively charged and aromatic group; π–π interactions: stacking of two aromatic groups face-to-face or face-to-edge; water bridges: distance of 2.8 Å between the donor and acceptor with ≥ 110° and ≥ 90° for donor and acceptor angles. Minimum distances between selected atom(s) and water were monitored by trajectory_asl_monitor.py script (Schrödinger LLC). Replica trajectories were merged before specific analysis using the trj_merge.py script (Schrödinger LLC). The output was transformed to csv-format by st2csv.py (Schrödinger LLC) and plotted by seaborn (v0.12.1)^87^.

Representative snapshots illustrating the putative binding modes were derived by utilizing the Trajectory Frame Clustering tool (trj_cluster.py script; Schrödinger LLC), using the key interaction residues (Figs. 2b, 3b): Lys16, Glu62, Asp69, His95 and Tyr96 for GDC-6036, Gly10, Glu62, Glu63, Asp69 and Tyr96 for LY3537982, for generating the RMSD matrix (fitted with protein backbone) that was used in the affinity propagation clustering method^88^, producing representative frames as output for each cluster, and the most populated cluster representing all the key interactions with the inhibitor was selected as the representative binding mode from the simulations. The 3D visualization of the predicted binding mode was conducted with PyMOL (The PyMOL Molecular Graphics System, Version 2.5.4 Schrödinger, LLC).

### WaterMap simulations

WaterMap^61,62^ simulations were conducted for the representative snapshot structures with all waters deleted, excluding the Mg^2+^-ion associated water molecules (see supplementary pdb-files). After energy minimization of these structures with Protein Preparation Wizard^79^ (see settings above), WaterMap simulations were carried out using the default parameters, which involved analyzing water molecules within a 10.0 Å radius of the SII-P bound ligand. The simulations were run for a duration of 2.0 ns. 3D illustrations of the WaterMap results were made with PyMOL (The PyMOL Molecular Graphics System, Version 2.5.4 Schrödinger, LLC).

### Nucleotide exchange assay

Nucleotide exchange assays using the QRET principle^63–65,68^ were performed in assay buffer containing 20 mM HEPES (pH 7.5) 1 mM MgCl_2_, 10 mM NaCl, 0.01% (v/v) Triton X-100. In assay, 10 nM KRAS(G12C), (G12C/H95L), (G12C/Y96D), and (WT), NRAS(G12C), HRAS(G12C)^68^ were preincubated with the covalent inhibitor, GDC-6036 and LY3537982 (0–30 μM) for 15 min in 6 μL volume. Thereafter, 6 μL of detection solution (3.5 μM MT2 and 10 nM Eu^3+^-GTP) was added and reaction was initiated with 5 nM SOS^cat^ added in 3 μL volume. TRL-signals were monitored using Tecan Spark 20 M from Tecan Life Sciences (Männedorf, Switzerland) by using 340 and 620 nm excitation and emission wavelengths and 800 and 400 μs delay and decay time, respectively. All concentrations are informed in a final 15 μL reaction volume in white 384-well low volume plates (Corning, USA). IC_50_ values were obtained using standard sigmoidal fitting functions from Origin 2016 software (Origin Lab, Northampton, MA).

### Thermal stability assay

FRET-Probe thermal stability assays^69^ were conducted in triplicates in an assay buffer containing 10 mM HEPES (pH 7.5), 10 mM NaCl, 0.5 mM MgCl_2_, and 0.01% (v/v) Brij-30. Assay was also performed in an assay buffer without MgCl_2_. 20 nM KRAS(G12C) and KRAS(WT) were preincubated with the covalent inhibitor, GDC-6036 and LY3537982 (0–30 μM) for 15 min in 12 μL volume. Thereafter, 8 μL of the 1 × FRET-Probe was added, and signals were monitored using step-by-step protocol by heating from 34–96 °C using 2 °C steps and heating the sample 1.5 min at each step prior measurement using 340 and 665 nm excitation and emission wavelengths and 50 and 200 μs delay and decay time, respectively. Thermal heating was performed with a PTC-100 Programmable Thermal Controller (MJ Research, Inc., Watertown, MA). All concentrations are informed in a final 20 μL reaction volume in white 96-well PCR-plate (BioRad, USA). *T*_m_ values were obtained using standard sigmoidal fitting functions from Origin 2016 software.

### Chemical stability assay

FRET-Probe chemical stability assays^69^ were performed in triplicates in an assay buffer containing 10 mM HEPES (pH 7.5), 10 mM NaCl, and 0.01% (v/v) Brij-30 (with or without 0.2 mM MgCl_2_) and destabilizing detection buffer with 10 mM HEPES (pH 7.5), 1 M urea, and 0.01% (v/v) Brij-30. 20 nM KRAS(G12C), (G12C/H95L), and (G12C/Y96D) were preincubated with the covalent inhibitor, GDC-6036 and LY3537982 (0–30 μM) for 15 min in 4 μL volume in assay buffer. Thereafter, 16 μL of the 1 × FRET-Probe was added in destabilizing detection buffer and TR-FRET signal was monitored multiple times during 120 min incubation (parameters as in thermal stability assay). All concentrations are informed in a final 20 μL reaction volume in white 384-well low volume plates (Corning, USA). IC_50_ values were obtained by blotting the signals obtained with different inhibitor concentrations at the selected time point against the inhibitor concentration and using standard sigmoidal fitting functions from Origin 2016 software.

### Origin of compounds and material

GDC-6036 was ordered from Chemgood LLC (Henrico, VA, USA) and LY3537982 was obtained from MedChemExpress, (Sollentuna, Sweden). *E. coli* expression, and purification of SOS^cat^ (564–1048); full-length KRAS(G12C), KRAS(G12C/H95L), KRAS(G12C/Y96D) and KRAS(WT); NRAS(12C) and HRAS(G12C) constructs (1–169); is described elsewhere^64,65,68^, and proteins were a kind gift from Leidos Biomedical Research, Inc., Frederick National Laboratory for Cancer Research and from University of Zurich. Eu^3+^-GTP and MT2 quencher were from QRET Technologies (Turku, Finland)^63,64,89,90^. FRET-Probe was designed and produced as described previously^69^.

## Electronic supplementary material

Below is the link to the electronic supplementary material.

Supplementary Material 1

Supplementary Material 2

Supplementary Material 3

Supplementary Material 4

Supplementary Material 5

Supplementary Material 6

Supplementary Material 7

Supplementary Material 8

## Data Availability

Raw trajectories of the Desmond molecular dynamics simulations and WaterMap results are freely available at: 10.5281/zenodo.8332412 (GDC-6036) and 10.5281/zenodo.8332200 (LY3537982).
