# Supplementary material for: Binding modes of the KRAS(G12C) inhibitors GDC-6036 and LY3537982 revealed by all atom molecular dynamics simulations
Source: Sci Rep. 2025 Jul 10;15:24843. doi: 10.1038/s41598-025-07532-2 (PMC12246139; doi:10.1038/s41598-025-07532-2)
Supplement: Supplementary file 8 — Supplementary Material 8 [file 41598_2025_7532_MOESM8_ESM.pdf]

Supporting Information

Binding modes of the KRAS(G12C) inhibitors GDC-6036 and LY3537982 revealed by all atom molecular dynamics simulations

Renne Leini[1], Jonas Kapp[2], Kari Kopra[3], Tatu Pantsar[1]*

[1]School of Pharmacy, Faculty of Health Sciences, University of Eastern Finland, Yliopistonrinne 3, 70210 Kuopio, Finland

[2]Department of Biochemistry, University of Zurich, Winterthurerstrasse 190, 8057 Zurich, Switzerland

[3]Department of Chemistry, University of Turku, Henrikinkatu 2, 20500 Turku, Finland

*E-mail: tatu.pantsar@uef.fi

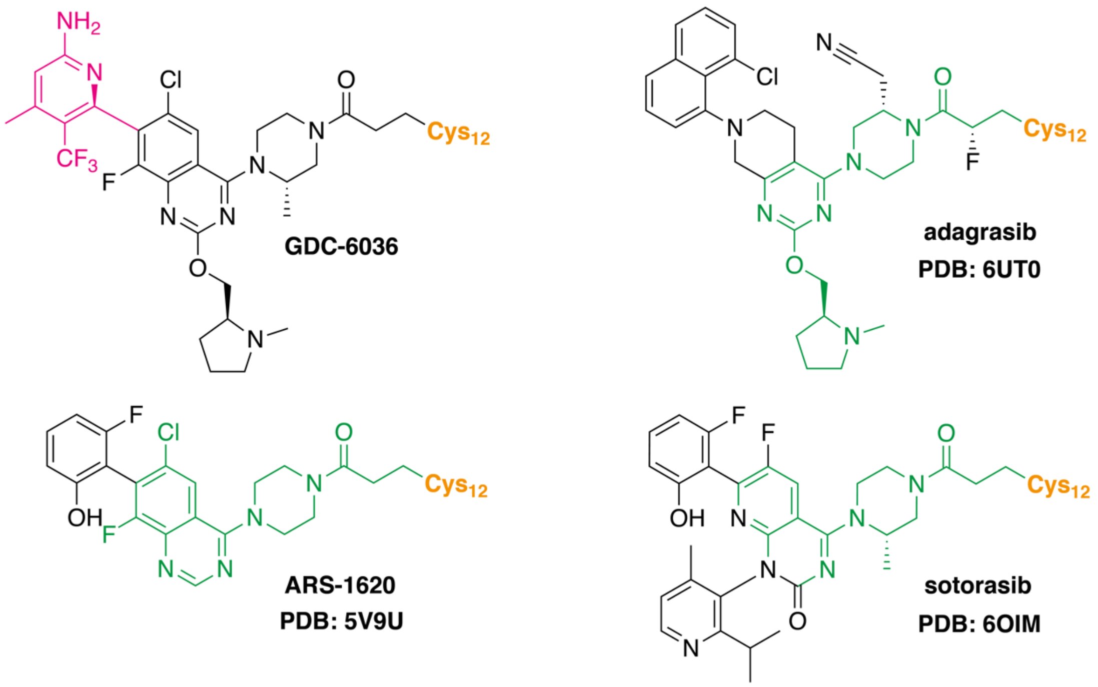

Figure S1. Analogous SII-P binders with GDC-6036 that exist with publicly available structural data. Only the moiety that is highlighted in red was not present in the publicly available KRAS co-crystal structures. The structural similarities of adagrasib, ARS-1620 and sotorasib with GDC-6036 are highlighted in green.

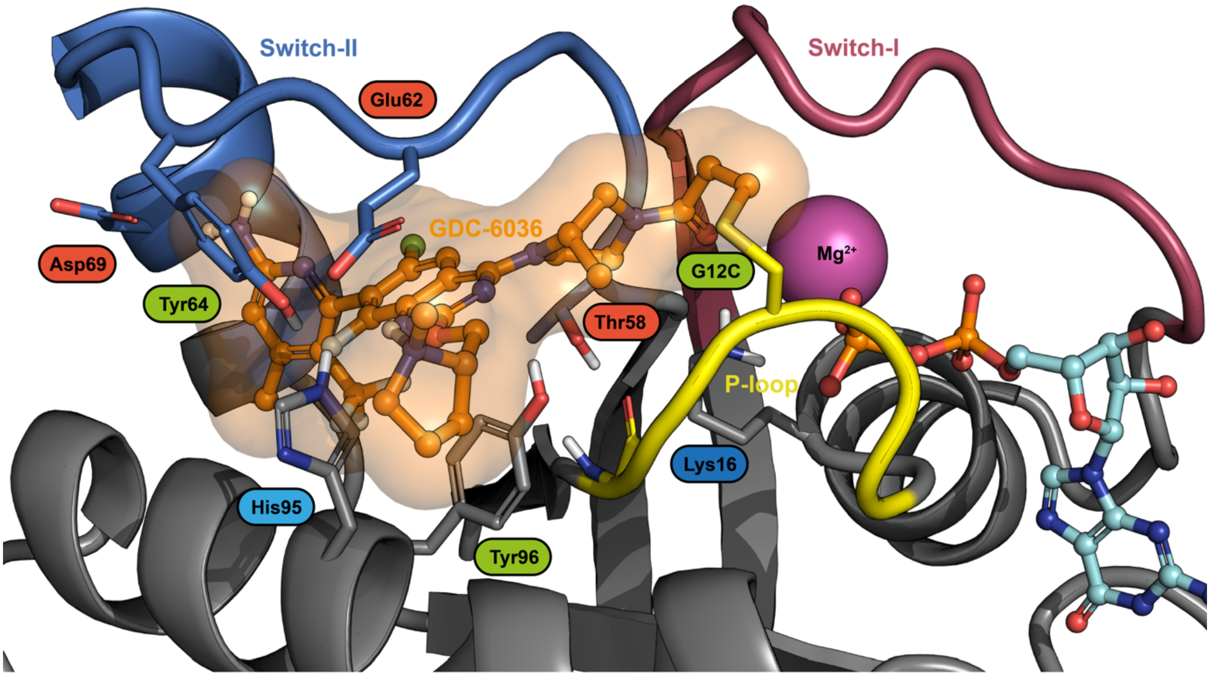

Figure S2. Starting configuration of the GDC-6036 simulations. The KRAS switch-II pocket from the first frame of the MD simulations is visualized using a cartoon representation. Selected key residues are highlighted as stick models. The ligand is represented using a ball-and-stick model with a transparent surface overlay.

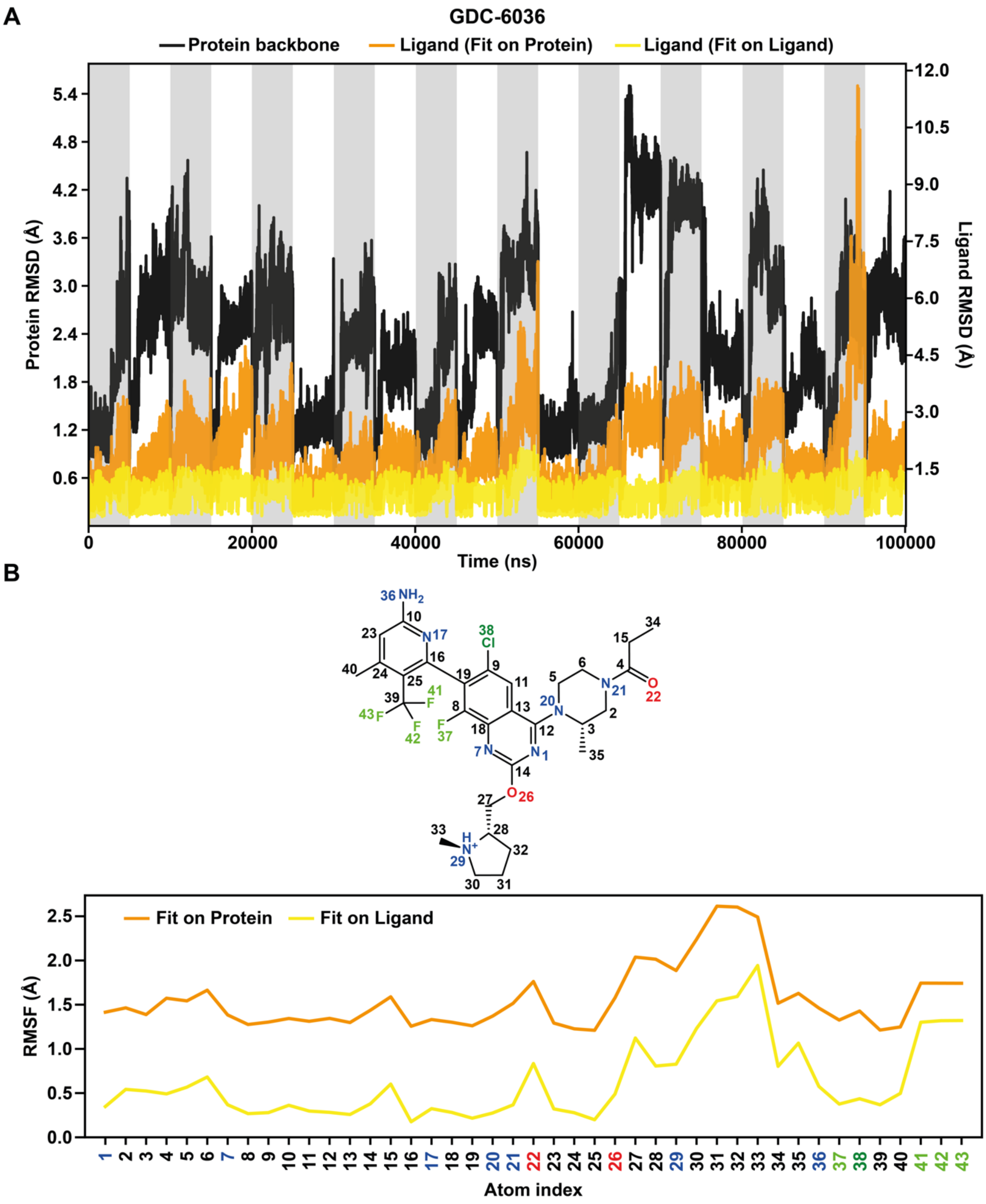

Figure S3. Protein and ligand root-mean-square deviation (RMSD) and ligand root-mean-square fluctuation (RMSF) plots from the GDC-6036 MD simulations. (**A**) Root-mean-square deviation (RMSD) of protein backbone and GDC-6036 (fitted on ligand and fitted on protein) in the simulations. The graph shows the concatenated trajectories of 20 replicas (each 5 μs long) analyzed at 1 ns intervals. (**B**) Root-mean-square fluctuation (RMSF) of GDC-6036 heavy atoms (fitted on ligand and fitted on protein) in the simulations.

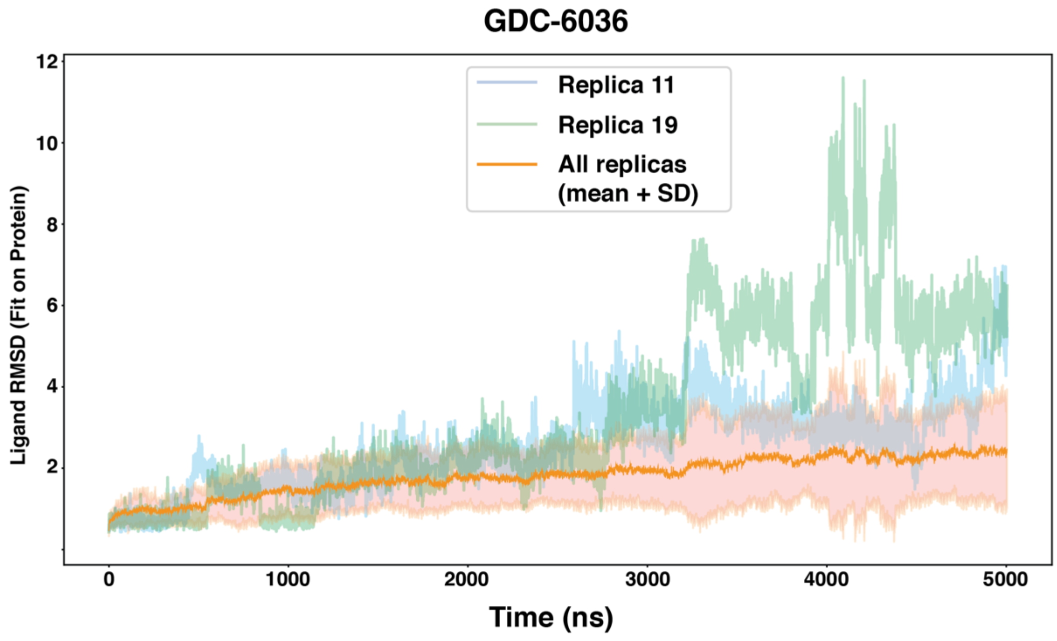

Figure S4. GDC-6036 simulations ligand RMSDs with potential outliers. Individual RMSD plots of the two replicas that display variable behavior compared to the other systems are shown together with RMSD plot of all systems (mean + SD). Data consist of 20 replicas (each 5 µs long) analyzed at 1 ns intervals.

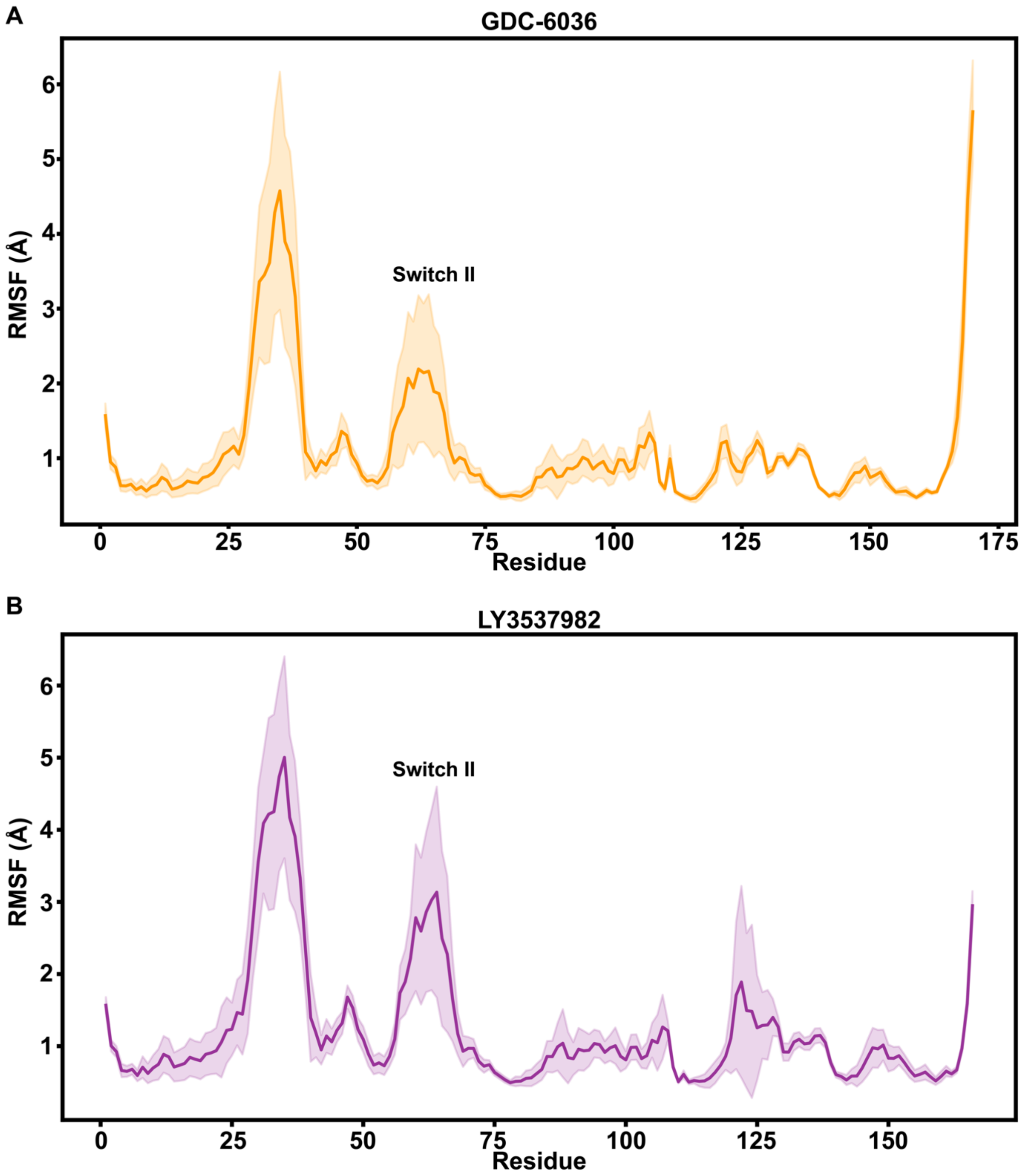

Figure S5. Protein backbone root-mean-square fluctuation (RMSF) plots of GDC-6036 and LY3537982 MD simulations. RMSF of protein backbone in simulations of (**A**) GDC-6036 and (**B**) LY3537982. Line represents the average of the 20 replicas and shaded area is the SD.

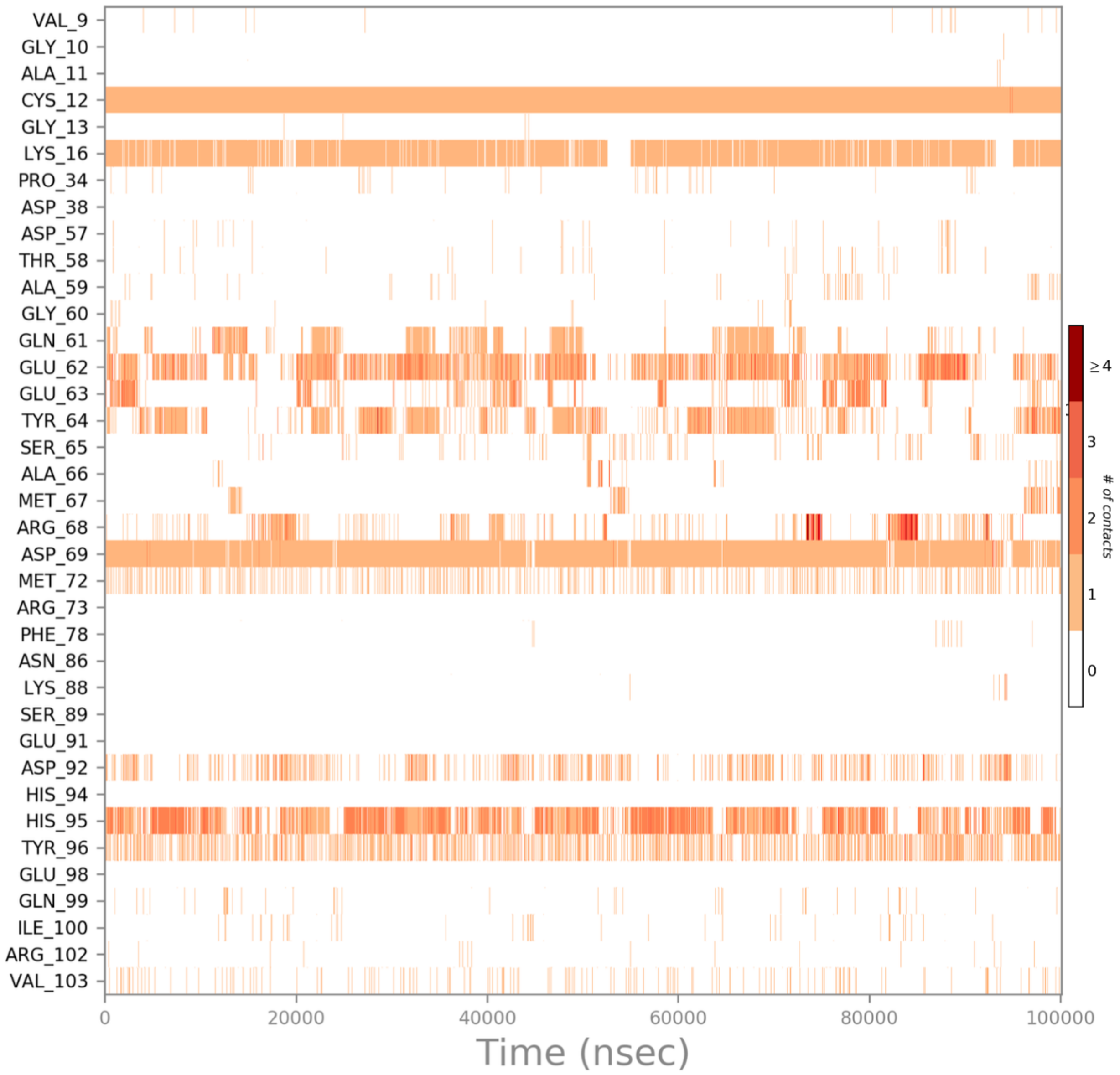

Figure S6. Timeline of the observed interactions in the MD simulations of GDC-6036. The observed key interactions are conserved across simulation replicas. The graph shows the concatenated trajectories of 20 replicas (each 5 μs long) analyzed at 1 ns intervals.

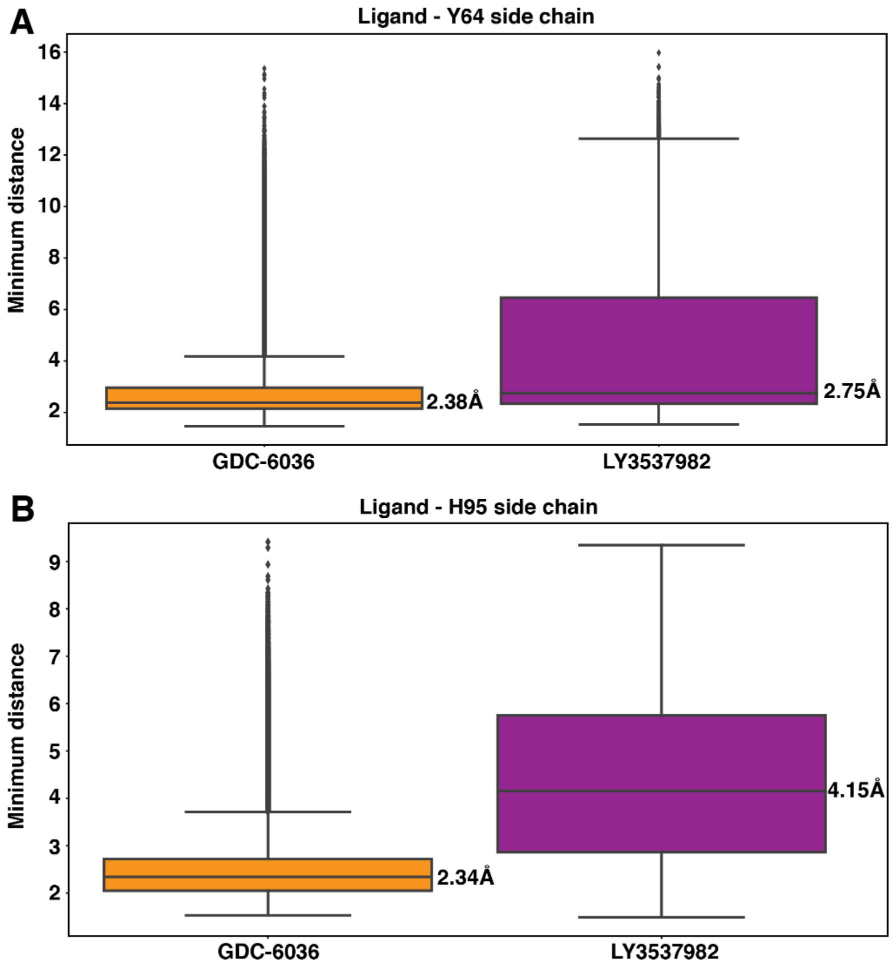

Figure S7. Minimum distance between the ligand and Tyr64 or His95 side chain in the simulations. The black horizontal line in the box represents the median. Box displays the quartiles of the dataset (25–75%) and whiskers the rest of the data with maximum 1.5 IQR. Outliers are indicated with black diamonds. The 100 μs simulation data of both systems was analyzed by 1 ns intervals.

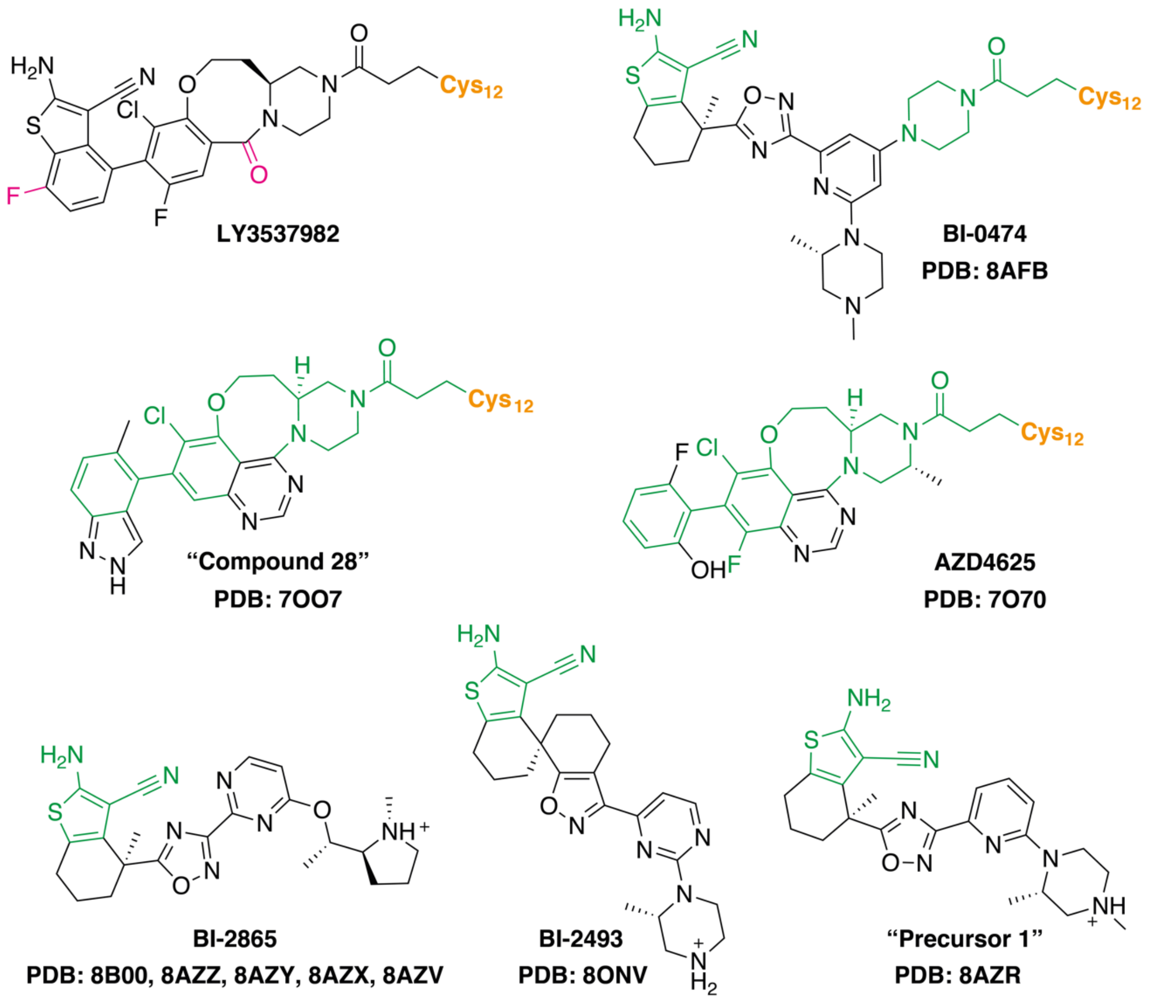

Figure S8. Analogous SII-P binders with LY3537982 that exist with publicly available structural data. Similar structural features that are highlighted in red were not present in the publicly available KRAS co-crystal structures. The structural similarities of BI-0474, "compound 28", AZD4625, BI-2865, BI-2493 and "precursor 1" with LY3537982 are highlighted in green.

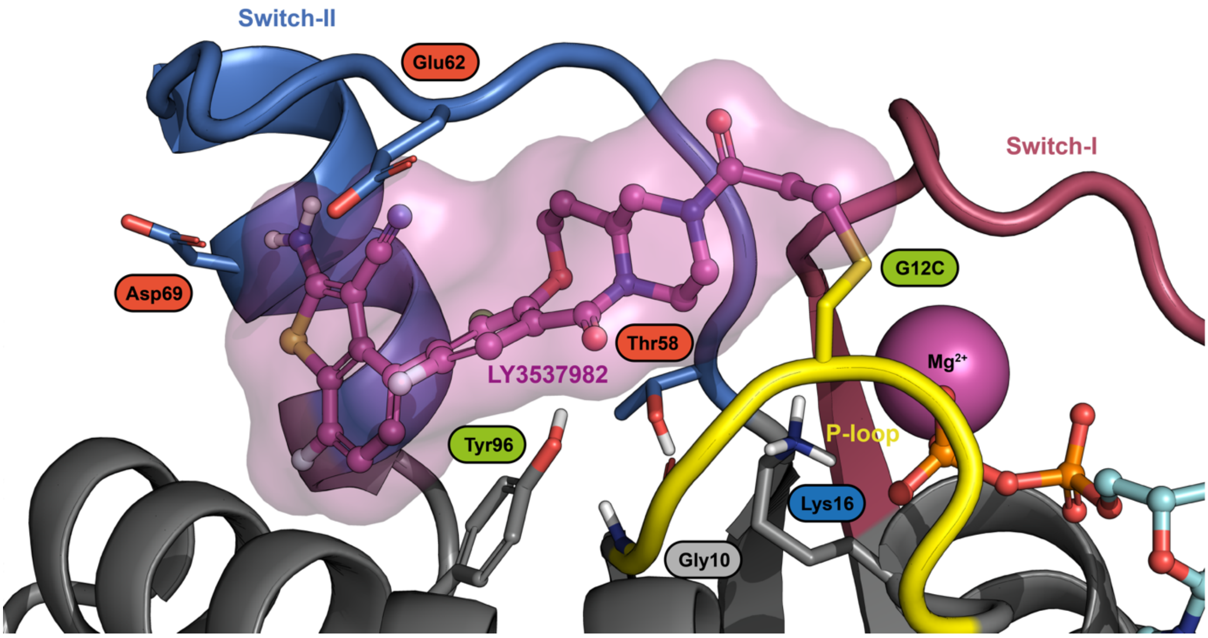

Figure S9. Starting configuration of the LY3537982 simulations. The KRAS switch-II pocket from the first frame of the MD simulations is visualized using a cartoon representation. Selected key residues are highlighted as stick models. The ligand is represented using a ball-and-stick model with a transparent surface overlay.

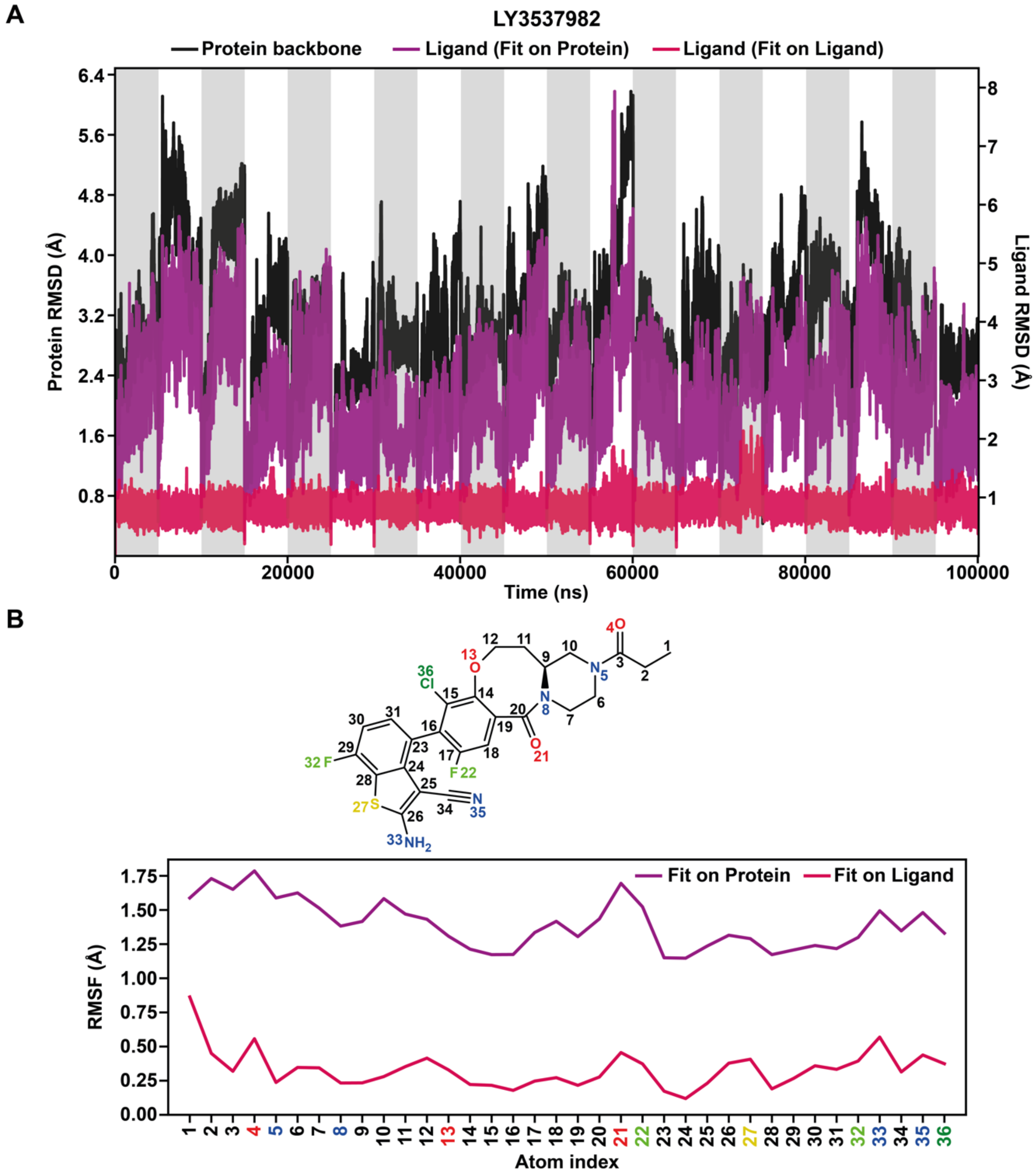

Figure S10. Protein and ligand root-mean-square deviation (RMSD) and ligand root-mean-square fluctuation (RMSF) plots from the LY3537982 MD simulations. (**A**) RMSD of protein backbone and LY3537982 (fitted on ligand and fitted on protein) in the simulations. The graph shows the concatenated trajectories of 20 replicas (each 5 μs long) analyzed at 1 ns intervals. (**B**) RMSF of LY3537982 heavy atoms (fitted on ligand and fitted on protein) in the simulations.

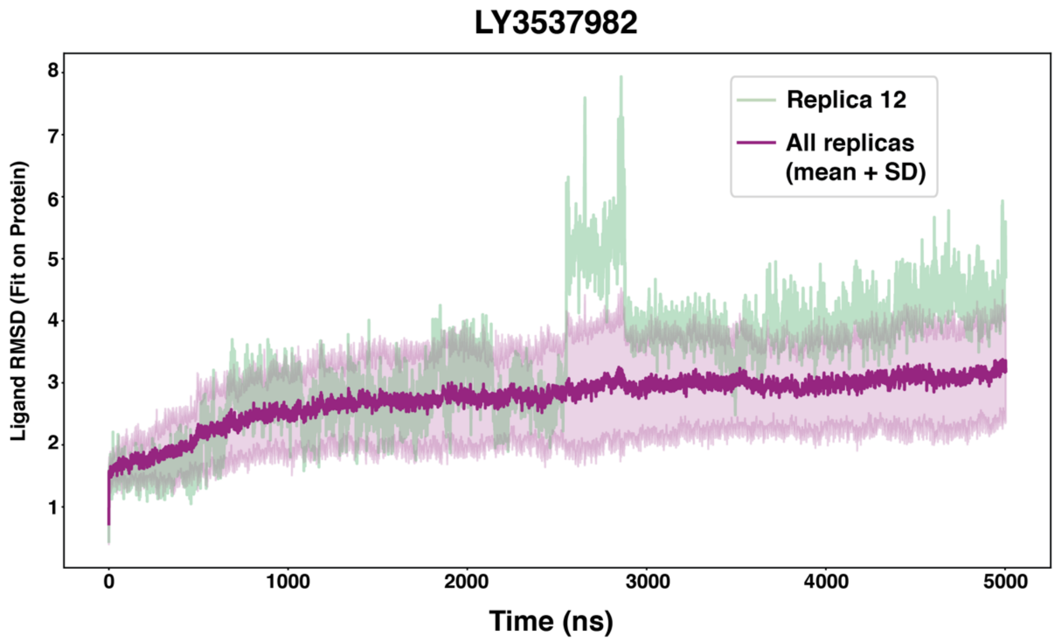

Figure S11. LY3537982 simulations ligand RMSDs with potential outliers. Individual RMSD plot of the replica that display variable behavior compared to the other systems are shown together with RMSD plot of all systems (mean + SD). Data consist of 20 replicas (each 5 µs long) analyzed at 1 ns intervals.

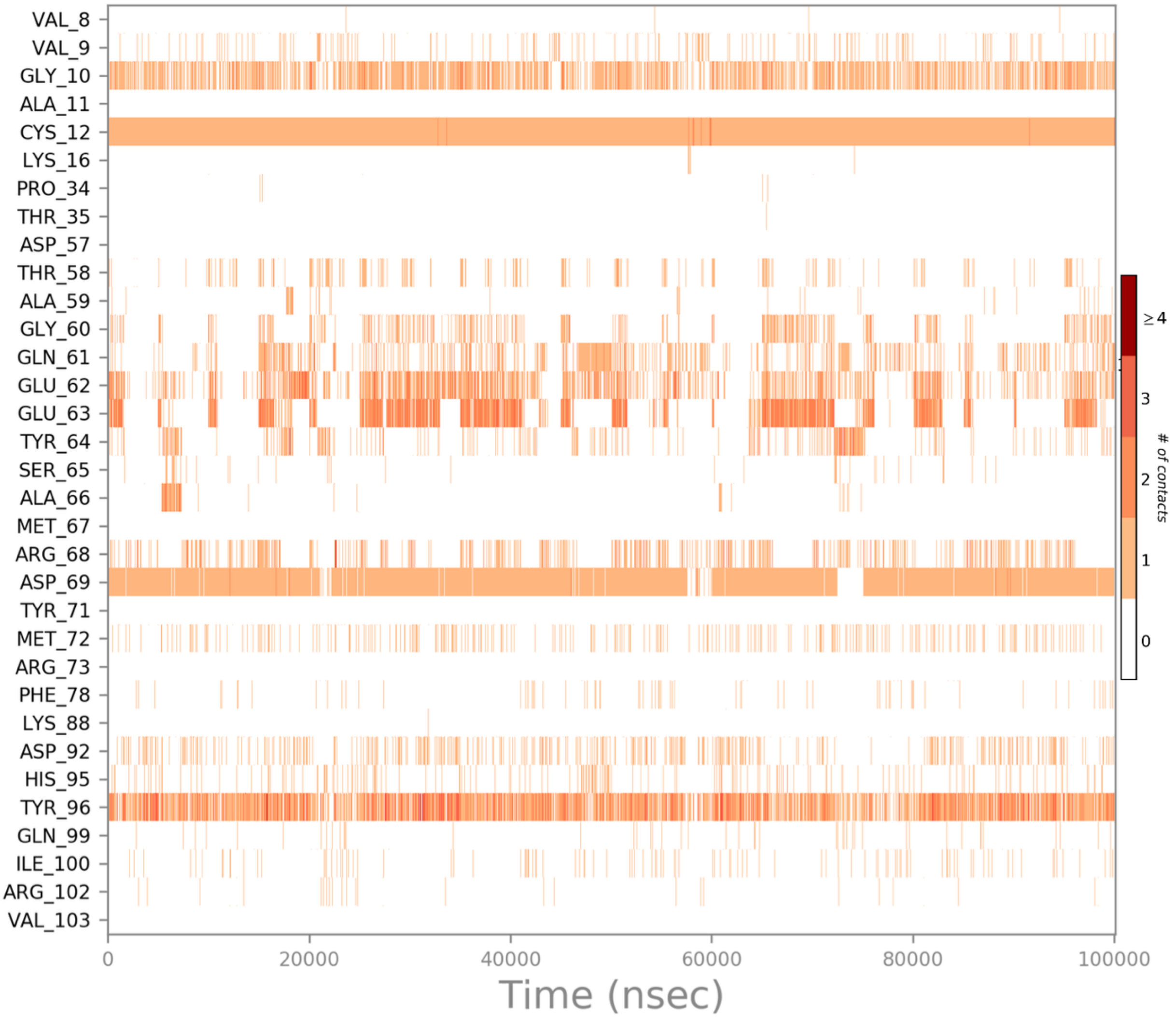

Figure S12. Timeline of the observed interactions in the MD simulations of LY3537982. The observed key interactions are conserved across simulation replicas. The graph shows the concatenated trajectories of 20 replicas (each 5 μs long) analyzed at 1 ns intervals.

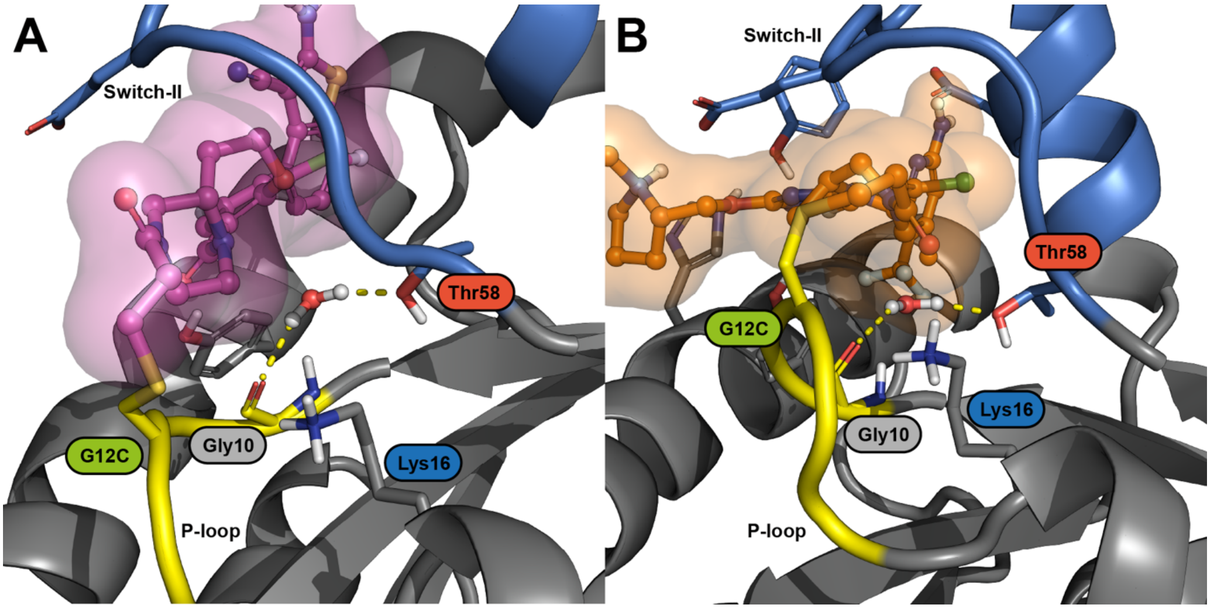

Figure S13. Thr-58 associated conserved water is present in the simulation starting configurations. In both systems (**A**: KRAS(G12C)–LY3537982; **B**: KRAS(G12C)–GDC-6036), the water molecule displays H-bond interactions to Thr58 and Gly10.

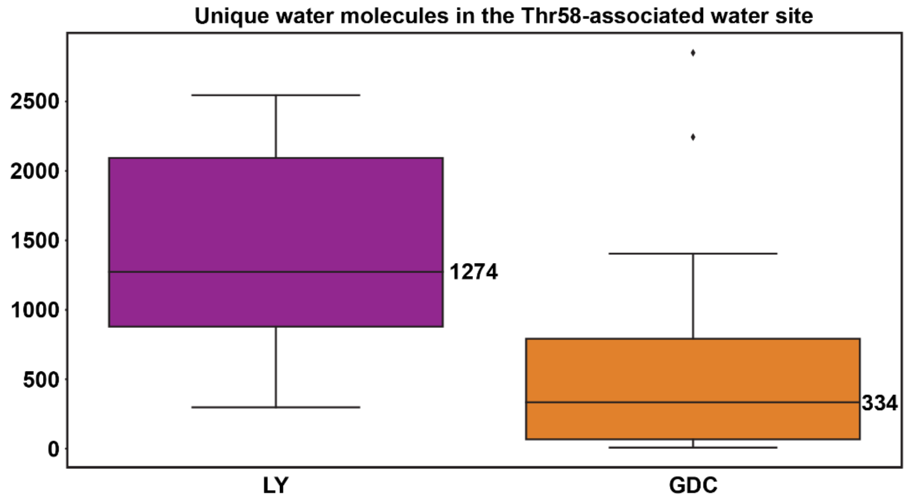

Figure S14. Unique water molecules observed in the Thr58-associated conserved water site. For each replica, the number of unique water molecule IDs were calculated using a 2 Å distance cutoff from the water site defined by WaterMap (see **Fig. S11**).

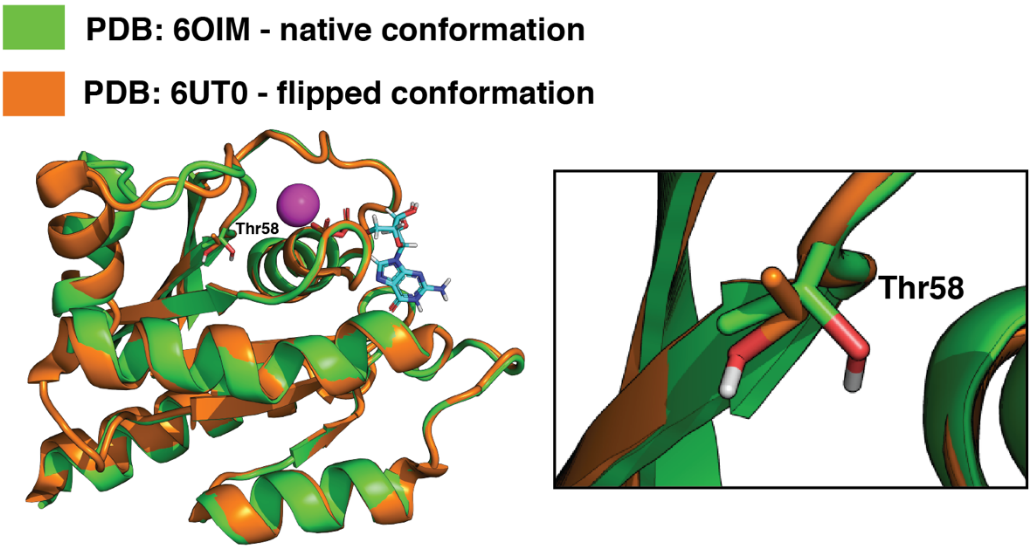

Figure S15. Thr58 orientation in KRAS crystal structures. An example of the typically observed "native" conformation of Thr58 is illustrated with the KRAS(G12C)-sotorasib co-crystal structure (6OIM) (sotorasib not show in the image). An example of the "flipped" conformation of Thr58 is illustrated with the KRAS(G12C)-adagrasib co-crystal structure (6UT0) (adagrasib not show in the image).

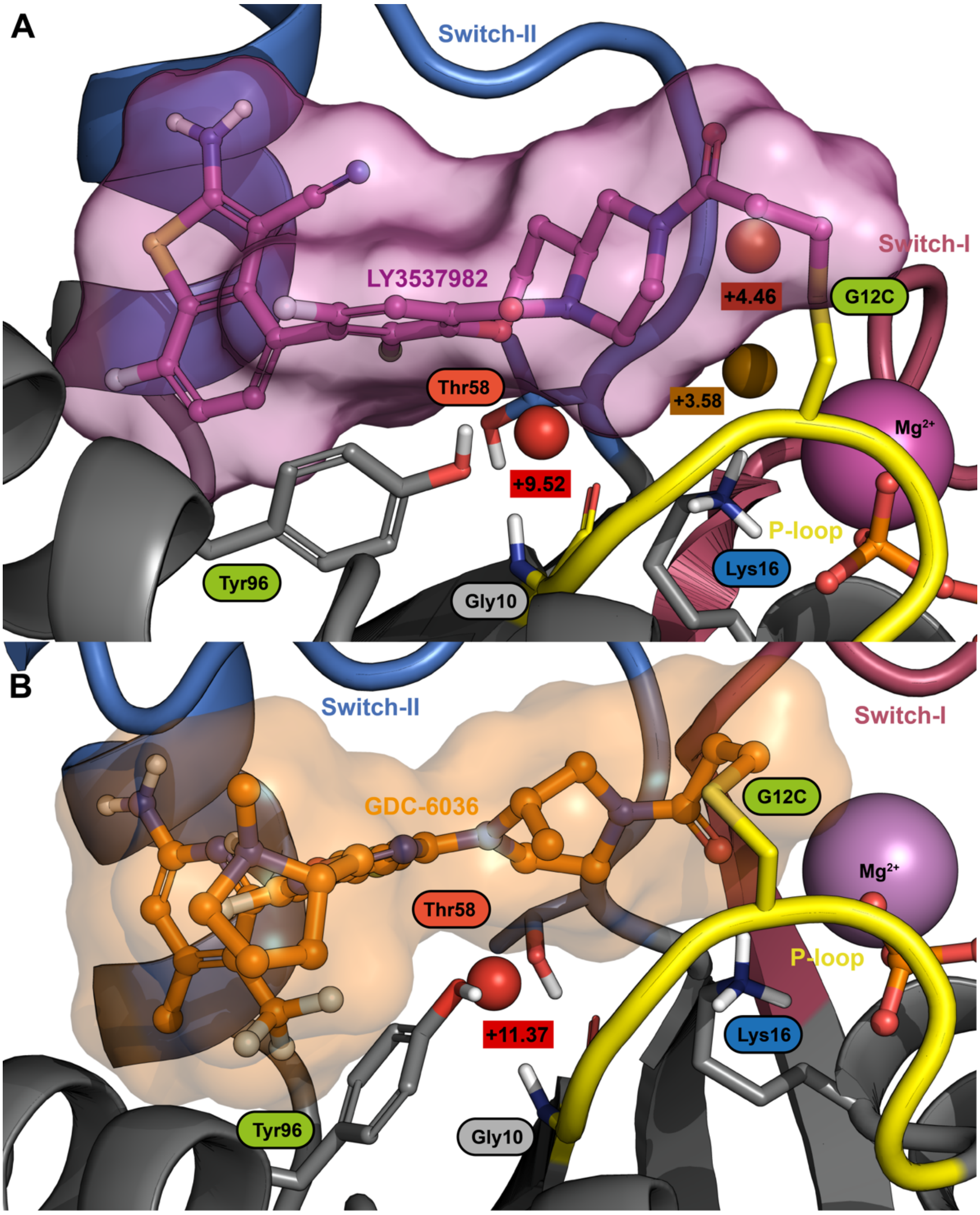

Figure S16. High energy WaterMap sites from the representative MD snapshots of GDC-6036 and LY3537982. WaterMap simulations suggest a high energy for the conserved water in the representative snapshots of (**A**) LY3537982 and (**B**) GDC-6036. Water energies in kcal/mol. For LY3537982, the water sites near the Lys16 are also shown (that are occupied by the carbonyl with GDC-6036).

Table S1. Occupancy of the conserved hydration site based on Thr58 sidechain conformation. Thr58 conformations were defined based on the distance between Thr58 sidechain O and Gly10(N)H and the angle formed by Thr58(O)–Thr58(Cβ)–Gly10(H). Conformation criteria were defined as follows: **native state**, distance: 3.6-4.8 Å, angle 5°-30°; **flipped state**: distance: 5.5-6.4 Å, angle 95°-120° (See Fig 4.).

Ligand	Thr58 native conformation water occupancy	Thr58 flipped conformation water occupancy	Time spent in the native conformation (%)	Time spent in the flipped conformation (%)
LY3537982	0.94	0.73	9.7	11.6
GDC-6036	0.57	0.03	26.4	7.6

Table S2. WaterMap results for the conserved water site. (HB = H-bonds; W = water; P = protein; L = ligand; energies in kcal/mol)

Ligand	Site	Occupancy	Overlap	ΔH	-TΔS	ΔG	#HB(WW)	#HB(PW/LW)
LY3537982	7	0.98	0	4.65	4.87	9.52	0	1.99
GDC-6036	6	0.97	0	7	4.37	11.37	0	1.7

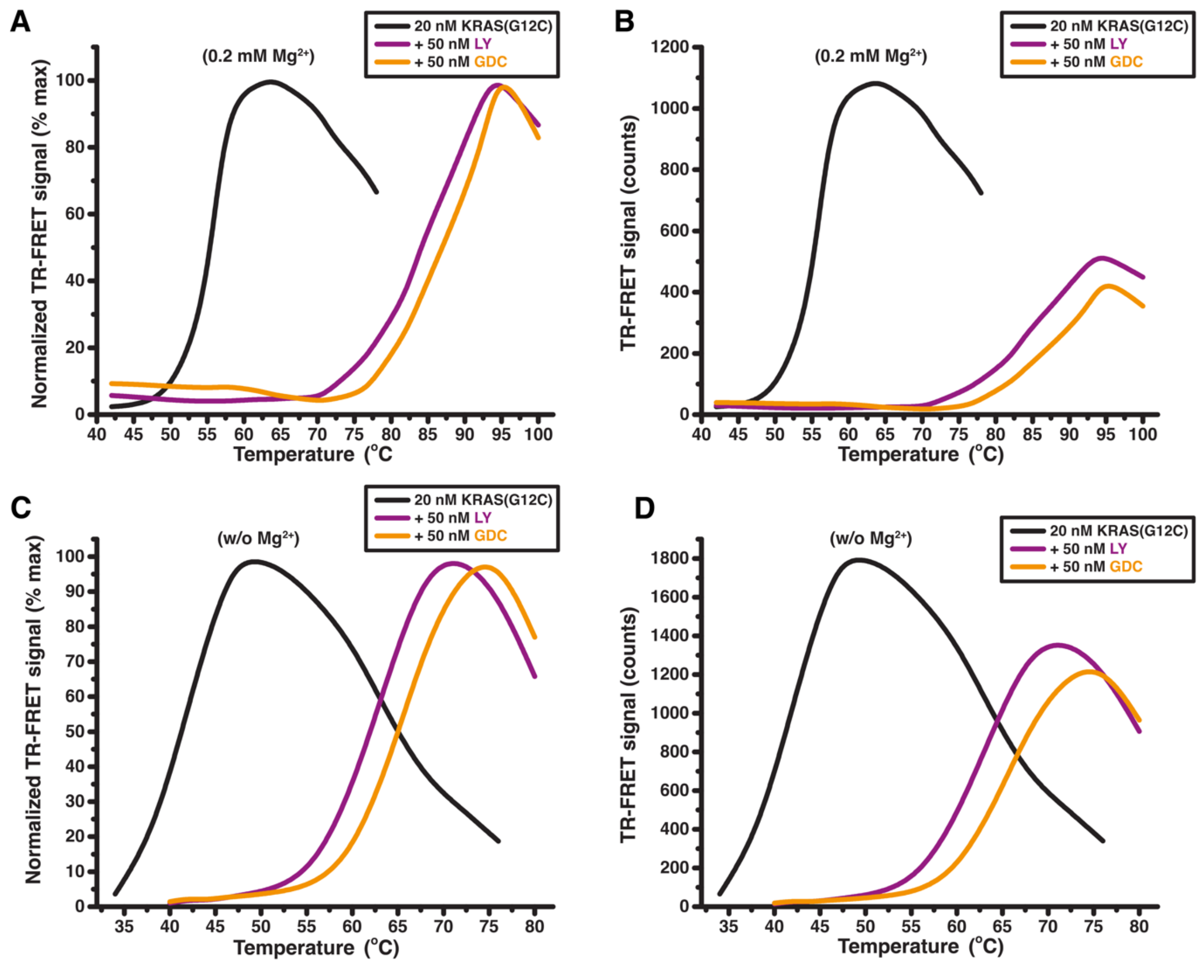

Figure S17. KRAS(G12C) thermal stability with GDC-6036 and LY3537982. KRAS(G12C) (20 nM) thermal stability was monitored using 50 nM GDC-6036 and LY3537982 in a step-by-step heating with the FRET-Probe. KRAS(G12C) was preincubated 15 min prior the thermal ramping, in which the TR-FRET signals from the triplicate reactions were monitored for every 2 °C, up to 96 °C. Normalized thermal curves with or without $MgCl_2$ (0.2 mM) are shown in **A** and **C**, and the same data is presented as TR-FRET signals in **B** and **D**, respectively.

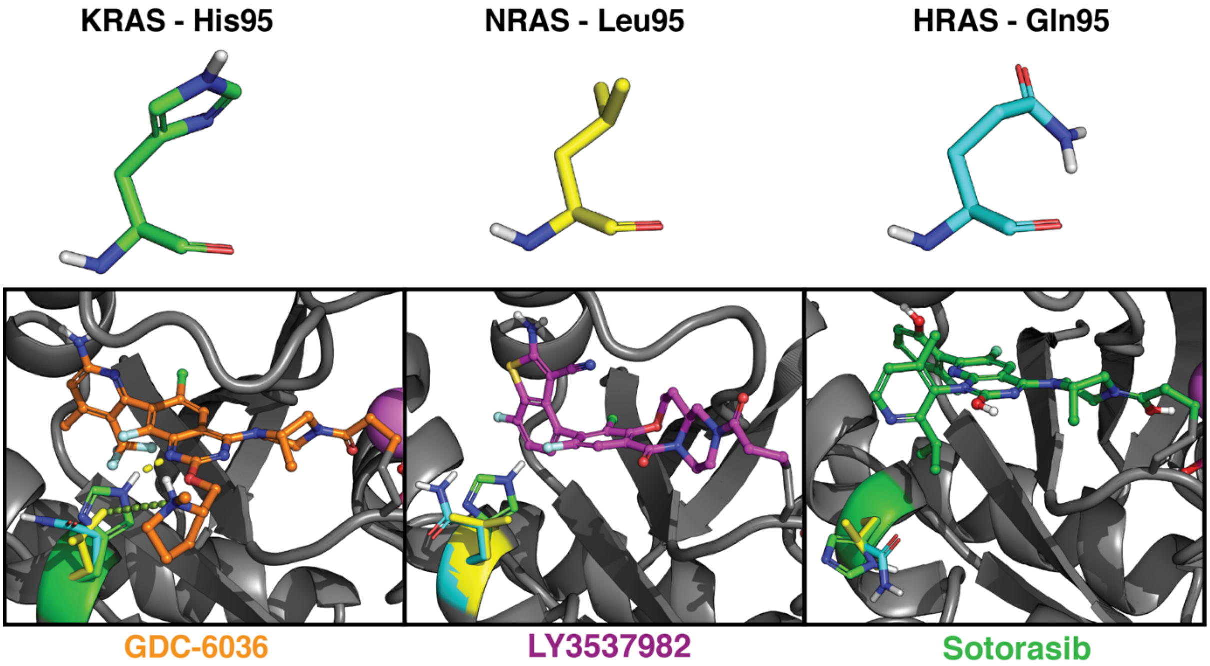

Figure S18. RAS isoform specific differences at position 95 and its location related to selected inhhibitors. KRAS contains a His at position 95, while Leu and Gln are observed with NRAS and HRAS, respectively. RAS isoform specific residue 95 is shown as stick models with the following color scheme: **KRAS**, green; **NRAS**, yellow; **HRAS**, cyan. Representative MD snapshots of the putative binding modes of GDC-6036 and LY3537982, and KRAS(G12C)-sotorasib structure (PDB: 6OIM) are represented as gray cartoon models. Ligands are shown as ball-and-stick models. Interactions from the residue 95 are highlighted: H-bonds, yellow dotted lines; cation–π interactions, green dotted lines. NRAS and HRAS model structures were generated by mutating and minimizing the residue 95 of the KRAS structures.

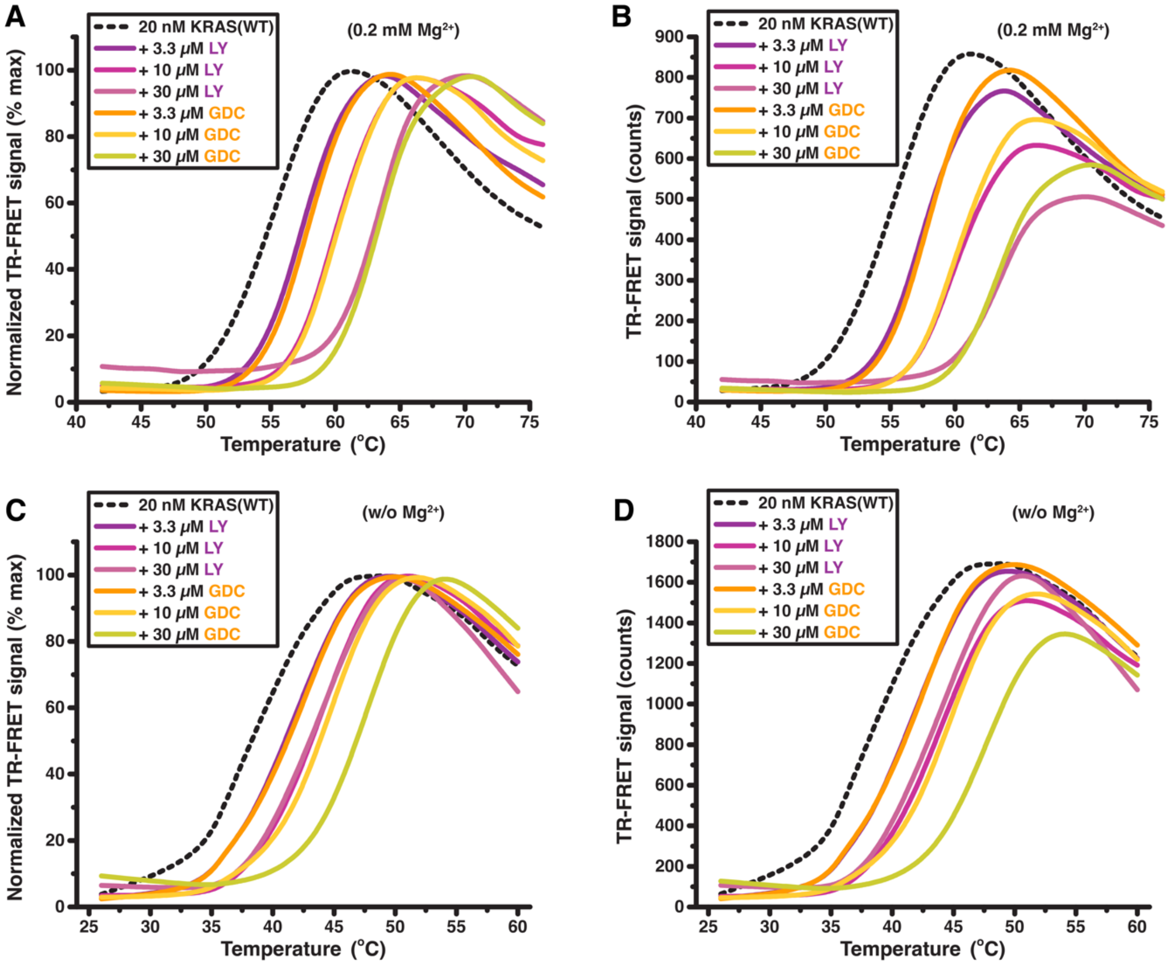

Figure S19. KRAS(WT) thermal stability with GDC-6036 and LY3537982. KRAS(G12C) (20 nM) thermal stability was monitored using 0-30 µM GDC-6036 and LY3537982 in a step-by-step heating with the FRET-Probe. KRAS(G12C) was preincubated 15 min prior the thermal ramping, in which the TR-FRET signals from the triplicate reactions were monitored for every 2 °C, up to 96 °C. Normalized thermal curves with or without $MgCl_2$ (0.2 mM) are shown in **A** and **C**, and the same data is presented as TR-FRET signals in **B** and **D**, respectively.

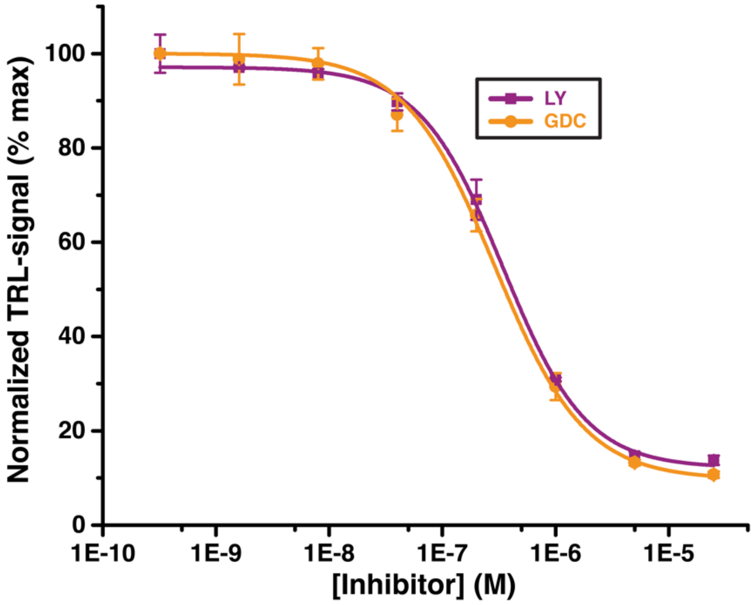

Figure S20. KRAS(WT) nucleotide exchange inhibition with GDC-6036 and LY3537982. Nucleotide exchange inhibition with GDC-6036 and LY3537982 (0-30 μM) was monitored in an assay with 10 nM KRAS(WT) and 5 nM SOS^{cat}, utilizing Eu^{3+}-GTP (10 nM) TRL-signal protection upon KRAS binding according to QRET principle. Data are displayed as mean ± SD.

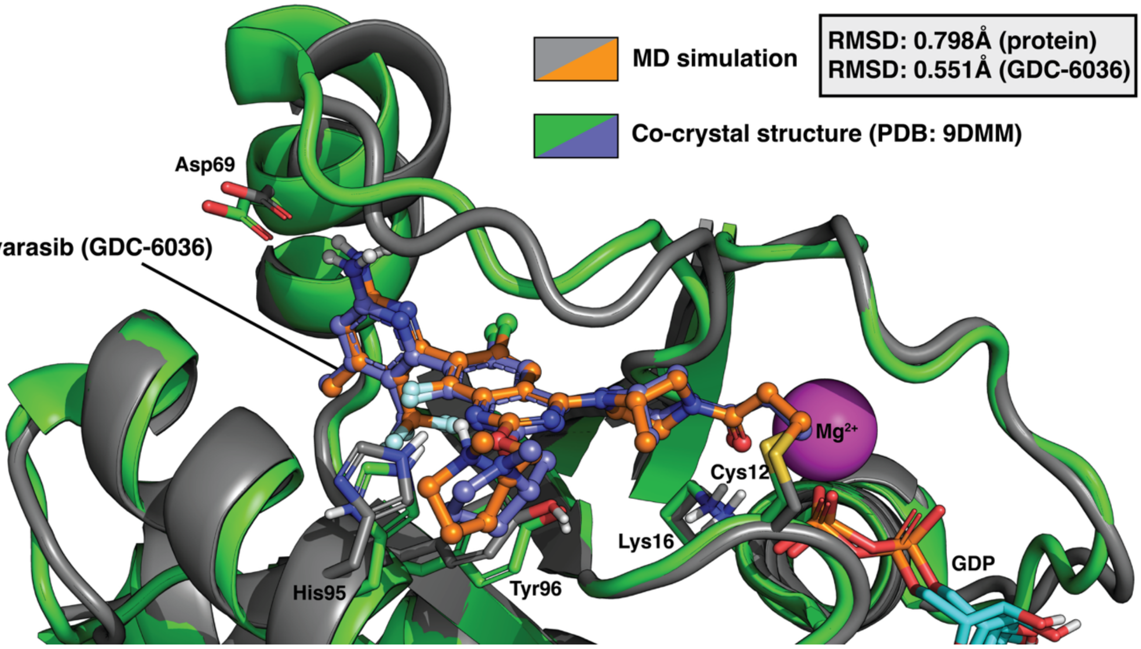

Figure S21. Comparison of MD predicted binding mode of GDC-6036 and its X-ray co-crystal structure. The prediction made by MD simulations is in good agreement with the experimental structure (PDB: 9DMM). After the superposition of the structures by their Cα atoms, RMSD values of 0.798 Å and 0.551 Å are observed for the protein (Cα atoms) and the heavy atoms of GDC-6036, respectively.
